# Elevated preoperative red cell distribution width and incident infection after metabolic and bariatric surgery: a cohort study

**DOI:** 10.3389/fnut.2026.1871866

**Published:** 2026-07-27

**Authors:** Ying-Jen Chang, Ping-Hsin Liu, Kuo-Mao Lan

**Affiliations:** 1 Department of Anesthesiology, Chi Mei Medical Center, Tainan City, Taiwan; 2 Department of Recreation and Health-Care Management, College of Recreation and Health Management, Chia Nan University of Pharmacy and Science, Tainan City, Taiwan; 3 Department of Anesthesiology, E-Da Hospital, I-Shou University, Kaohsiung City, Taiwan; 4 Department of Anesthesiology, Chi Mei Hospital, Liouying, Tainan City, Taiwan

**Keywords:** metabolic and bariatric surgery, perioperative risk stratification, propensity score matching, red cell distribution width, sepsis

## Abstract

**Introduction:**

Red cell distribution width (RDW), an index of erythrocyte size variability associated with inflammation and nutritional status, may have prognostic utility; however, its role in predicting infection after metabolic and bariatric surgery (MBS) remains unclear.

**Methods:**

This TriNetX retrospective cohort study included adults undergoing primary MBS from 2010–2025. Elevated preoperative RDW was defined as ≥14.6% across all measurements within 3 months before surgery, while normal RDW ranged from 11.5 to 14.5%. Patients with discordant values were excluded. One-to-one propensity score matching was performed. The primary outcome was incident sepsis within 1 year, and secondary outcomes included pneumonia, surgical site infection (SSI), urinary tract infection (UTI), and intensive care unit (ICU) admission.

**Results:**

After matching, 17,735 patients were included in each group. Elevated RDW was associated with an increased risk of sepsis (hazard ratios [HRs] 1.42, 95% confidence interval [CI] 1.15–1.75; *p* = 0.001), pneumonia (HR 1.33; *p* = 0.003), and ICU admission (HR 1.22; *p* = 0.018), but not UTI or SSI. Temporal analysis showed that the risk of pneumonia was highest early postoperatively, whereas the risk of sepsis increased over time. These associations persisted across surgical subtypes and were not fully explained by baseline anemia. Elevated RDW was also associated with subsequent iron deficiency (ferritin< 30 ng/mL) and inflammation markers (C-reactive protein >10 mg/L).

**Conclusion:**

Elevated preoperative RDW is associated with distinct temporal patterns of increased 1-year sepsis and pneumonia risks after MBS. RDW may serve as a simple, low-cost biomarker for perioperative risk stratification. Further prospective studies are warranted.

## 1 Introduction

Metabolic and bariatric surgery (MBS) is the most effective treatment for severe obesity (1, 2). However, postoperative infectious complications, including sepsis, pneumonia, and surgical site infection, remain clinically important adverse events after MBS and are associated with increased postoperative morbidity, mortality, and resource utilization (3, 4). The identification of preoperative biomarkers associated with infection risk could enable more tailored perioperative optimization and risk-adapted postoperative surveillance. Red cell distribution width (RDW), a routinely reported component of the complete blood count, quantifies the heterogeneity of circulating erythrocyte size (5). Originally used in the differential diagnosis of anemia, RDW has increasingly been recognized as an integrative hematologic marker influenced by systemic inflammation, oxidative stress, and nutritional deficiencies (6, 7). Elevated RDW has been associated with adverse outcomes after cardiovascular and general surgery, as well as with mortality in critically ill patients with sepsis (8–12). In the bariatric population, however, prior investigations have focused exclusively on the relationship between RDW and postoperative weight loss (13, 14); whether preoperative RDW elevation predicts infectious complications after MBS has not been examined. This retrospective cohort study evaluated the association between elevated preoperative RDW and incident infection within one year after MBS, using propensity score–matched data from a large multinational federated database.

## 2 Methods

### 2.1 Data source and ethics

This retrospective cohort study used de-identified electronic health record data from the TriNetX Global Collaborative Network, a federated platform comprising 173 healthcare organizations across multiple countries. The TriNetX platform has been widely used in peer-reviewed real-world evidence studies (15–17) and has supported hundreds of published observational analyses. Because all data were aggregated and HIPAA-compliant, the Institutional Review Board approved the protocol and waived the requirement for informed consent. All procedures performed in studies involving human participants were in accordance with the ethical standards of the institutional and/or national research committee and with the 1964 Helsinki Declaration and its later amendments or comparable ethical standards.

### 2.2 Study population and exposure definition

Adults (≥18 years) who underwent primary metabolic and bariatric procedures between January 1, 2010, and December 31, 2025, were identified using CPT codes 43,775 (laparoscopic sleeve gastrectomy) and 43,644 (laparoscopic Roux-en-Y gastric bypass). The index date was defined as the date of the surgery. The preoperative RDW was assessed using all available RDW-CV measurements reported as percentages (%) within 3 months before surgery. Multiple RDW measurements were not required for inclusion; patients with a single eligible preoperative RDW measurement were classified according to that value. For patients with more than one RDW measurement during the preoperative window, all available values were required to fall within the same RDW category. Patients were classified as having elevated RDW if all available values were ≥14.6% and as having normal RDW if all values were 11.5–14.5%; those with discordant values were excluded to reduce exposure misclassification. This approach was used to create mutually exclusive exposure groups and to avoid assigning patients with fluctuating RDW values to either cohort based on a single potentially nonrepresentative measurement. Consistent with commonly reported clinical laboratory reference intervals for RDW, values of 11.5–14.5% were treated *a priori* as the normal reference range in this study; accordingly, ≥14.6% represented the first value above this upper limit and provided a clinically interpretable, prespecified threshold for elevated RDW.

To minimize confounding from pre-existing critical illness, end-organ failure, or unrelated immunocompromise, patients were excluded if they had a diagnosis of chronic kidney disease stage 4–5, end-stage renal disease, dialysis dependence, or HIV infection at any time before the index date, or if they had severe sepsis, other sepsis, acute kidney failure, COVID-19, influenza/pneumonia, or critical-care services within one month before surgery. Patients who received same-day perioperative transfusion were excluded because transfusion both alters hematologic measurements and independently increases infection risk, thereby confounding the relationship between preoperative RDW and subsequent infection. Complete code-level definitions are provided in Supplementary material 1.

### 2.3 Outcomes

The primary outcome was incident sepsis (severe sepsis, other sepsis, or streptococcal sepsis) within 365 days after surgery. Secondary clinical outcomes included pneumonia, surgical site infection (superficial or deep incisional), urinary tract infection, and ICU admission. ICU admission was defined using the TriNetX standardized procedure concept for critical care services and should be interpreted as ICU-level care or critical care service use. Planned postoperative ICU monitoring and unplanned ICU admission could not be reliably distinguished in the structured aggregate data. Patients were followed from the index date until the occurrence of the outcome of interest, death, or the end of the 365-day follow-up period, whichever occurred first. To probe the biological coherence of the exposure, three hematologic and inflammatory biomarkers were also assessed during follow-up: persistent RDW elevation (>14.5%), iron depletion (ferritin <30 ng/mL), and systemic inflammation (CRP ≥ 10 mg/L). To explore temporal patterns, all outcomes were additionally analyzed within three discrete post-index intervals: 1–30 days, 31–180 days, and 181–365 days.

### 2.4 Covariates and propensity score matching

Baseline covariates spanned four domains: demographics (age, sex, race, BMI category), comorbidities (e.g., diabetes, iron- and B12-deficiency anemia, neoplasms, liver disease, alcohol- and nicotine-related disorders), preoperative laboratory indices (hemoglobin, albumin, HbA1c, ferritin, CRP, leukocyte count, eGFR, vitamin B12), and concomitant medications (insulin, GLP-1 receptor agonists, SGLT2 inhibitors, oral/parenteral iron, systemic corticosteroids). These variables were selected based on their established or biologically plausible roles in systemic inflammation, iron metabolism, glycaemic burden, and erythropoiesis, which are mechanistically linked to the pathway between RDW and infection risk. One-to-one propensity score matching was performed with a greedy nearest-neighbor algorithm using a caliper of 0.1 standard deviations of the logit of the propensity score. Balance was assessed by absolute standardized mean differences (SMDs), with an SMD < 0.10 considered adequate.

### 2.5 Statistical analysis

Time-to-event associations were estimated using Cox proportional hazards regression and reported as hazard ratios (HRs) with 95% confidence intervals. The proportional hazards assumption was tested using Schoenfeld residuals. Schoenfeld residual tests were reported for each Cox model. If the primary outcome violated the proportional hazards assumption, time-varying or interval-specific estimates were considered. Exploratory secondary and biomarker outcomes with non-proportional hazards were interpreted cautiously, with interval-specific analyses used descriptively. For the primary outcome, an E-value was computed to quantify the minimum strength of association that an unmeasured confounder would require to nullify the observed effect (18). A pre-specified subgroup analysis was performed by surgical procedure (sleeve gastrectomy vs. Roux-en-Y gastric bypass), and a sensitivity analysis was performed by baseline anemia status, each repeating propensity score matching within strata. For the surgical subgroup analyses, patients were restricted to each surgical procedure subgroup before propensity score matching was repeated; therefore, the matched sample sizes in the subgroup analyses were generated independently and were not expected to sum to the sample size of the primary matched cohort. Baseline anemia was operationally defined using pre-index ICD-10-CM diagnosis codes for anemia, including D64 (other anemias) and D50–D53 (nutritional anemias), rather than laboratory hemoglobin thresholds. Patients were stratified according to the presence or absence of these diagnosis codes before the index date. To address temporal and measurement-related sources of bias, we performed two additional sensitivity analyses: one restricted to surgeries performed during the contemporary ICD-10 coding era (2016–2025) and another restricted to patients with an RDW measurement within 1 week before surgery. Each analysis repeated propensity score matching and Cox proportional hazards modeling.

Statistical significance was set at a two-sided *p* < 0.05 for the primary outcome; secondary, subgroup, and biomarker analyses were considered exploratory and reported without adjustments for multiple comparisons. Missing laboratory data were not imputed. Patients were not excluded solely because ancillary baseline laboratory variables were unavailable, and the availability of laboratory data before and after matching is available in the Supplementary material 1. All analyses were performed using the TriNetX analytics platform.

## 3 Results

### 3.1 Baseline characteristics and primary outcomes

A total of 21,089 patients with elevated preoperative RDW (≥14.6%) and 57,995 patients with normal preoperative RDW (11.5–14.5%) were included. Before matching, the elevated-RDW group differed substantially from the comparator group in terms of race, iron deficiency anemia (SMD = 0.437), preoperative hemoglobin <12 g/dL (SMD = 0.590), and ferritin <30 ng/mL (SMD = 0.430) (Table 1). After 1:1 propensity score matching, 17,735 patients remained in each group, with all covariates achieving an absolute standardized mean difference (SMD) below 0.10. Baseline laboratory availability before and after matching is summarized in Supplementary material 1; after matching, availability was highest for hemoglobin and eGFR and lowest for CRP.

**Table 1:** Baseline characteristics of patients undergoing metabolic and bariatric surgery.

Variables	Before matching			After matching		
	Elevated RDW group (*n* = 21,089)	Control group (*n* = 57,995)	SMD	Elevated RDW group (*n* = 17,735)	Control group (*n* = 17,735)	SMD
Patient characteristics						
Age at index (years)	43.5 ± 11.9	43.2 ± 11.8	0.027	44.2 ± 11.9	44.1 ± 11.9	0.025
Female	18,311 (86.8)	46,821 (80.7)	0.166	15,062 (84.9)	15,089 (85.1)	0.004
BMI 35–40 (kg/m^2^)	5,139 (24.4)	19,524 (33.7)	0.206	4,555 (25.7)	4,421 (24.9)	0.017
BMI 40–45 (kg/m^2^)	8,292 (39.3)	27,672 (47.7)	0.170	7,157 (40.4)	6,927 (39.1)	0.027
BMI 45–50 (kg/m^2^)	7,808 (37.0)	20,667 (35.6)	0.029	6,514 (36.7)	6,450 (36.4)	0.007
White	10,262 (48.7)	40,150 (69.2)	0.428	9,528 (53.7)	9,403 (53.0)	0.014
Black or African American	8,054 (38.2)	10,072 (17.4)	0.478	5,808 (32.7)	5,967 (33.6)	0.019
Asian	131 (0.6)	351 (0.6)	0.002	112 (0.6)	104 (0.6)	0.006
Comorbidities						
Essential hypertension	13,481 (63.9)	33,293 (57.4)	0.134	11,324 (63.9)	11,473 (64.7)	0.018
Sleep apnea	13,241 (62.8)	34,203 (59.0)	0.078	11,189 (63.1)	11,282 (63.6)	0.011
Other anxiety disorders	7,912 (37.5)	23,771 (41.0)	0.071	6,841 (38.6)	6,831 (38.5)	0.001
Diabetes mellitus	8,093 (38.4)	17,642 (30.4)	0.168	6,760 (38.1)	6,770 (38.2)	0.001
Diseases of liver	5,162 (24.5)	15,105 (26.0)	0.036	4,457 (25.1)	4,406 (24.8)	0.007
Neoplasms	4,306 (20.4)	10,763 (18.6)	0.047	3,600 (20.3)	3,628 (20.5)	0.004
Iron deficiency anemia	4,390 (20.8)	3,604 (6.2)	0.437	2,602 (14.7)	2,563 (14.5)	0.006
Depressive disorder	2,448 (11.6)	6,631 (11.4)	0.005	2098 (11.8)	2026 (11.4)	0.013
Nicotine dependence	2,122 (10.1)	6,069 (10.5)	0.013	1796 (10.1)	1826 (10.3)	0.006
Ischemic heart diseases	1960 (9.3)	4,093 (7.1)	0.082	1,656 (9.3)	1720 (9.7)	0.012
COPD	1,079 (5.1)	1974 (3.4)	0.085	901 (5.1)	946 (5.3)	0.011
CKD	1,032 (4.9)	1852 (3.2)	0.086	860 (4.8)	843 (4.8)	0.004
Cerebrovascular diseases	564 (2.7)	1,138 (2.0)	0.047	463 (2.6)	487 (2.7)	0.008
Alcohol related disorders	426 (2.0)	1,184 (2.0)	0.002	369 (2.1)	370 (2.1)	0.000
Vitamin B12 deficiency anemia	178 (0.8)	401 (0.7)	0.017	145 (0.8)	159 (0.9)	0.009
Laboratory data						
Hemoglobin≥12 g/dL	16,491 (78.2)	56,194 (96.9)	0.590	15,750 (88.8)	15,965 (90.0)	0.039
Albumin ≥ 3.5 g/dL	18,546 (87.9)	51,938 (89.6)	0.051	15,679 (88.4)	15,673 (88.4)	0.001
HbA1c ≥ 7%	3,346 (15.9)	7,089 (12.2)	0.105	2,822 (15.9)	2,815 (15.9)	0.001
Vitamin B12 ≤ 300 pg./mL	2,268 (10.8)	5,856 (10.1)	0.022	1859 (10.5)	1829 (10.3)	0.006
Ferritin < 30 ng/mL	5,546 (26.3)	5,854 (10.1)	0.430	3,659 (20.6)	3,735 (21.1)	0.011
eGFR≥60 mL/min/1.73 m^2^	19,501 (92.5)	54,701 (94.3)	0.075	16,432 (92.7)	16,447 (92.7)	0.003
CRP ≥ 10 mg/L	1887 (8.9)	3,410 (5.9)	0.117	1,497 (8.4)	1,523 (8.6)	0.005
Leukocytes≥ 11 × 10^3^/μ	5,579 (26.5)	13,934 (24.0)	0.056	4,659 (26.3)	4,594 (25.9)	0.008
Medications						
Insulins and analogues	6,257 (29.7)	13,758 (23.7)	0.135	5,225 (29.5)	5,267 (29.7)	0.005
Iron supplementation	4,757 (22.6)	5,219 (9.0)	0.379	3,096 (17.5)	2,998 (16.9)	0.015
GLP-1 analogues	3,171 (15.0)	8,004 (13.8)	0.035	2,666 (15.0)	2,645 (14.9)	0.003
SGLT2 inhibitors	877 (4.2)	1715 (3.0)	0.065	751 (4.2)	748 (4.2)	0.001
Type of surgery						
Sleeve surgery	14,362 (68.2)	37,445 (64.6)	0.078	11,976 (67.5)	12,068 (68.0)	0.010
Bypass surgery	6,727 (31.8)	20,550 (35.4)	0.075	5,759 (32.5)	5,667 (32.0)	0.011

Data are presented as *n* (%) for categorical variables and mean ± standard deviation for continuous variables. Absolute standardized mean differences (SMDs) were used to assess covariate balance; an SMD of <0.10 indicated adequate balance after matching. BMI, body mass index; CKD, chronic kidney disease stages 1–3; COPD, chronic obstructive pulmonary disease; HbA1c, hemoglobin A1c; eGFR, estimated glomerular filtration rate; CRP, C-reactive protein; GLP-1, glucagon-like peptide-1; SGLT2, sodium–glucose cotransporter 2; RDW, red cell distribution width.

**Figure 1:**
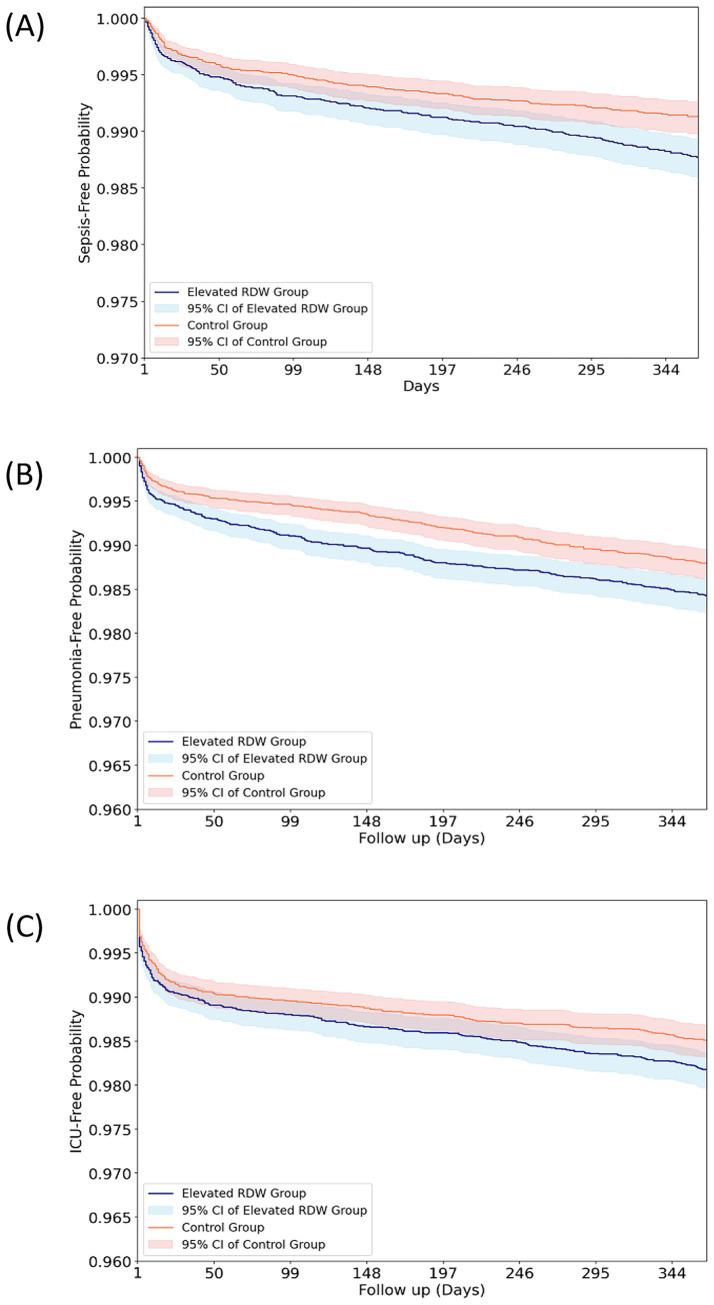
Kaplan–Meier curves for 1-year event-free probability in patients with elevated versus normal preoperative red cell distribution width (RDW) after metabolic and bariatric surgery. **(A)** Sepsis-free probability, **(B)** pneumonia-free probability, and **(C)** intensive care unit (ICU) admission-free probability in the propensity score–matched cohort. The elevated-RDW group showed lower event-free probability than the normal-RDW group for all three outcomes during the 1-year follow-up. Shaded areas indicate 95% confidence intervals. RDW, red cell distribution width; ICU, intensive care unit.

In the matched cohort, elevated preoperative RDW was associated with a higher 1-year risk of incident sepsis (HR 1.42, 95% CI 1.15–1.75; *p* = 0.001), pneumonia (HR 1.33; *p* = 0.003), and ICU admission (HR 1.22; *p* = 0.018) (Table 2). No significant association was observed between the occurrence of urinary tract infection (*p* = 0.898) or surgical site infection (*p* = 0.837). When expressed as absolute risks, the corresponding risk differences were modest, with excess events of 3.5 per 1,000 patients for sepsis, 3.7 per 1,000 for pneumonia, and 3.2 per 1,000 for ICU admission in the elevated-RDW group. Larger absolute differences were observed for biomarker outcomes, particularly persistent RDW elevation and ferritin <30 ng/mL (Table 3). Kaplan–Meier analysis showed a lower 1-year sepsis-free probability in the elevated RDW group than in the comparator group (98.77% vs. 99.13%; log-rank *p* = 0.001; Figure 1A), with curves progressively separating after the first postoperative month. Additional Kaplan–Meier curves for pneumonia (Figures 1B) and ICU admission (Figures 1C) similarly showed lower event-free probabilities in the elevated RDW group during the 1-year follow-up. The E-value was 2.19 for the point estimate and 1.57 for the lower 95% CI, suggesting moderate robustness against unmeasured confounding. All three biomarker outcomes, including persistent RDW elevation, ferritin <30 ng/mL, and CRP ≥ 10 mg/L, were associated with elevated preoperative RDW (*p* < 0.001).

**Table 2:** Association between elevated preoperative red cell distribution width and 1-year clinical outcomes.

Outcome	Elevated RDW group (*n* = 17,735)	Control group (*n* = 17,735)	HR (95% CI)	*p*-value	*p* for proportionality
	Events (%)	Events (%)			
Clinical outcomes					
Sepsis (primary)	209 (1.18%)	147 (0.83%)	1.42 (1.15–1.75)	0.001	0.151
Pneumonia	267 (1.51%)	201 (1.13%)	1.33 (1.10–1.59)	0.003	0.071
UTI	769 (4.34%)	761 (4.29%)	1.01 (0.91–1.11)	0.898	0.021
SSI	42 (0.24%)	40 (0.23%)	1.05 (0.68–1.61)	0.837	0.780
ICU admission	312 (1.76%)	255 (1.44%)	1.22 (1.04–1.44)	0.018	0.308
Hematologic and inflammatory biomarkers					
RDW > 14.5%	15,045 (84.83%)	4,472 (25.22%)	6.53 (6.31–6.76)	<0.001	<0.001
Ferritin <30 ng/mL	3,010 (16.97%)	1,975 (11.14%)	1.57 (1.48–1.66)	<0.001	0.059
CRP ≥ 10 mg/L	646 (3.64%)	495 (2.79%)	1.30 (1.16–1.47)	<0.001	0.028

CI, confidence interval; HR, hazard ratio; RDW, red cell distribution width; UTI, urinary tract infection; SSI, surgical site infection; ICU, intensive care unit; CRP, C-reactive protein. *p* for proportionality was derived from Schoenfeld residual tests. Values <0.05 indicate evidence against the proportional hazards assumption. Secondary clinical and biomarker outcomes were exploratory; outcomes with evidence of non-proportionality were interpreted cautiously and further described in interval-specific analyses.

**Table 3:** Absolute risks and risk differences per 1,000 matched patients.

Outcome	Elevated RDW group, events per 1,000 patients	Control group, events per 1,000 patients	Absolute risk difference per 1,000 patients
Sepsis	11.8	8.3	3.5
Pneumonia	15.1	11.3	3.7
UTI	43.4	42.9	0.5
SSI	2.4	2.3	0.1
ICU admission	17.6	14.4	3.2
RDW >14.5%	848.3	252.2	596.2
Ferritin <30 ng/mL	169.7	111.4	58.4
CRP ≥10 mg/L	36.4	27.9	8.5

Risk difference was calculated as the absolute risk in the elevated RDW group minus the absolute risk in the control group. RDW, red cell distribution width; UTI, urinary tract infection; SSI, surgical site infection; ICU, intensive care unit; CRP, C-reactive protein.

### 3.2 Temporal analysis

When the 1-year window was partitioned (Table 4), the association between elevated preoperative RDW and incident sepsis was not significant in the first month (HR 1.23; *p* = 0.227) but emerged at 1–6 months (HR 1.47; *p* = 0.024) and was most pronounced at 6–12 months (HR 1.72; *p* = 0.004). Pneumonia showed the opposite temporal pattern, with associations evident in the first month (HR 1.49; *p* = 0.010) and at 1–6 months (HR 1.63; *p* = 0.002) but attenuated at 6–12 months (*p* = 0.758). The association with ICU admission strengthened over time, reaching significance only at 6–12 months (HR 1.59; *p* = 0.009).

**Table 4:** Temporal analysis of the association between elevated preoperative red cell distribution width and infection outcomes across three postoperative intervals.

Outcomes	1 month^†^		1–6 month^†^		6–12 month^†^	
	HR (95% CI)	*p* value	HR (95% CI)	*p* value	HR (95% CI)	*p* value
Sepsis	1.23 (0.88–1.73)	0.227	1.47 (1.05–2.06)	0.024	1.72 (1.19–2.51)	0.004
Pneumonia	1.49 (1.10–2.01)	0.010	1.63 (1.20–2.20)	0.002	1.05 (0.77–1.42)	0.758
UTI	1.09 (0.88–1.34)	0.450	1.06 (0.92–1.22)	0.428	0.96 (0.82–1.12)	0.582
SSI	0.88 (0.50–1.56)	0.659	NA	NA	NA	NA
ICU admission	1.11 (0.89–1.38)	0.346	1.33 (0.97–1.82)	0.080	1.59 (1.12–2.25)	0.009
RDW > 14.5%	11.30 (10.73–11.90)	<0.001	2.99 (2.87–3.12)	<0.001	4.78 (4.48–5.10)	<0.001
Ferritin <30 ng/mL	1.65 (1.16–2.36)	0.005	1.62 (1.50–1.74)	<0.001	1.51 (1.41–1.63)	<0.001
CRP ≥ 10 mg/L	1.09 (0.91–1.31)	0.354	1.46 (1.22–1.74)	<0.001	1.35 (1.09–1.67)	0.006

^†^*n* = 17,735 for each group; CI, confidence interval; HR, hazard ratio; RDW, red cell distribution width; UTI, urinary tract infection; SSI, surgical site infection; ICU, intensive care unit; CRP, C-reactive protein; NA: not available due to insufficient number of events.

### 3.3 Subgroup analysis by type of surgery

The association between elevated preoperative RDW and incident sepsis was observed in both the sleeve gastrectomy (HR, 1.49; *p* = 0.010) and Roux-en-Y gastric bypass (HR, 1.63; *p* = 0.003) subgroups, with no evidence of effect modification (*p* for interaction = 0.689) (Table 5). No interaction was detected for any other clinical outcome (all *p* for interaction > 0.05). The only statistically significant interaction was observed for CRP ≥ 10 mg/L, where the association was confined to the sleeve subgroup (HR 1.44; *p* < 0.001) but absent in the bypass subgroup (HR 1.10; *p* = 0.276; *p* for interaction = 0.020).

**Table 5:** Subgroup analysis by surgical type.

Outcomes	Sleeve surgery^†^		Bypass surgery^‡^		*p* for interaction
	HR (95% CI)	*p* value	HR (95% CI)	*p* value	
Sepsis	1.49 (1.12–1.98)	0.01	1.63 (1.18–2.25)	0.003	0.689
Pneumonia	1.26 (1.00–1.60)	0.05	1.26 (0.95–1.68)	0.113	1.000
UTI	1.07 (0.94–1.22)	0.32	1.04 (0.89–1.22)	0.587	0.786
SSI	0.76 (0.42–1.37)	0.36	1.14 (0.64–2.05)	0.66	0.381
ICU admission	1.24 (1.00–1.55)	0.06	1.34 (1.05–1.72)	0.017	0.651
RDW > 14.5%	6.75 (6.47–7.05)	<0.001	6.33 (5.99–6.68)	<0.001	0.068
Ferritin <30 ng/mL	1.51 (1.40–1.63)	<0.001	1.48 (1.36–1.60)	<0.001	0.724
CRP ≥ 10 mg/L	1.44 (1.24–1.67)	<0.001	1.10 (0.93–1.31)	0.276	0.020

^†^*n* = 12,295 for each group; ^‡^*n* = 6,011 for each group; CI, confidence interval; HR, hazard ratio; RDW, red cell distribution width; UTI, urinary tract infection; SSI, surgical site infection; ICU, intensive care unit; CRP, C-reactive protein. *p* for interaction reflects the between-subgroup difference in log-hazard ratios; identical rounded HRs may yield *p* ≈ 1.000.

### 3.4 Sensitivity analysis stratified by baseline Anemia status

To assess whether the observed associations were driven primarily by underlying anemia rather than RDW-related erythropoietic stress, we repeated the primary and secondary analyses separately in patients with and without baseline anemia. Among the 4,571 matched pairs with baseline anemia, elevated RDW remained significantly associated with incident sepsis (HR, 1.79; *p* = 0.001) and ICU admission (HR, 1.63; *p* = 0.001) (Table 6). Among the 13,296 matched pairs without baseline anemia (Table 7), the association with pneumonia persisted (HR, 1.33; *p* = 0.015), whereas the association with sepsis was directionally consistent but attenuated and did not reach statistical significance (HR, 1.26; *p* = 0.094). These findings suggest that the prognostic value of elevated preoperative RDW, particularly for pneumonia, is not fully explained by the presence of anemia.

**Table 6:** Sensitivity analysis of association between elevated preoperative red cell distribution width and 1-year outcomes in patients with baseline anemia.

Outcome	Elevated RDW group (*n* = 4,571)	Control group (*n* = 4,571)	HR (95% CI)	*p*-value
	Events (%)	Events (%)		
Sepsis (primary)	82 (1.79%)	46 (1.01%)	1.79 (1.25–2.56)	0.001
Pneumonia	91 (1.99%)	69 (1.51%)	1.32 (0.97–1.81)	0.079
UTI	274 (5.99%)	284 (6.21%)	0.96 (0.82–1.14)	0.662
SSI	25 (0.55%)	13 (0.28%)	1.92 (0.98–3.76)	0.052
ICU admission	125 (2.74%)	77 (1.69%)	1.63 (1.23–2.16)	0.001
RDW > 14.5%	4,107 (89.85%)	1,492 (32.64%)	5.99 (5.63–6.37)	<0.001
Ferritin <30 ng/mL	1,076 (23.54%)	767 (16.78%)	1.48 (1.35–1.62)	<0.001
CRP ≥ 10 mg/L	245 (5.36%)	169 (3.70%)	1.46 (1.20–1.77)	<0.001

CI, confidence interval; HR, hazard ratio; RDW, red cell distribution width; UTI, urinary tract infection; SSI, surgical site infection; ICU, intensive care unit; CRP, C-reactive protein.

**Table 7:** Sensitivity analysis of association between elevated preoperative red cell distribution width and 1-year outcomes in patients without baseline anemia.

Outcome	Elevated RDW group (*n* = 13,296)	Control group (*n* = 13,296)	HR (95% CI)	*p*-value
	Events (%)	Events (%)		
Sepsis (primary)	121 (0.91%)	96 (0.72%)	1.26 (0.96–1.64)	0.094
Pneumonia	174 (1.31%)	131 (0.99%)	1.33 (1.06–1.66)	0.015
UTI	509 (3.83%)	474 (3.56%)	1.07 (0.95–1.22)	0.273
SSI	20 (0.15%)	17 (0.13%)	1.17 (0.62–2.24)	0.627
ICU admission	181 (1.36%)	170 (1.28%)	1.06 (0.86–1.31)	0.575
RDW > 14.5%	11,091 (83.42%)	3,086 (23.21%)	6.77 (6.50–7.05)	<0.001
Ferritin <30 ng/mL	1,895 (14.25%)	1,258 (9.46%)	1.54 (1.44–1.66)	<0.001
CRP ≥ 10 mg/L	417 (3.14%)	345 (2.60%)	1.21 (1.05–1.39)	0.010

CI, confidence interval; HR, hazard ratio; RDW, red cell distribution width; UTI, urinary tract infection; SSI, surgical site infection; ICU, intensive care unit; CRP, C-reactive protein.

### 3.5 Analyses restricted by coding era and preoperative RDW measurement window

In the 2016–2025 cohort, elevated RDW remained associated with sepsis (HR 1.55, *p* = 0.002), pneumonia (HR 1.29, *p* = 0.029), and ICU admission (HR 1.31, *p* = 0.014) (Table 8). Similarly, among patients with RDW measured within 1 week before surgery, elevated RDW was associated with sepsis (HR 1.59, *p* = 0.003), pneumonia (HR 1.32, *p* = 0.045), and ICU admission (HR 1.38, *p* = 0.004). No significant associations were observed for UTI or SSI in either sensitivity analysis.

**Table 8:** Sensitivity analyses of 1-year outcomes restricted to the contemporary ICD-10 era and to patients with RDW measured within 1 week before surgery.

Outcomes	Contemporary ICD-10 era, 2016–2025		RDW measured within 1 week before surgery	
	HR (95% CI)	*p* value	HR (95% CI)	*p* value
Sepsis	1.55 (1.17–2.05)	0.002	1.59 (1.17–2.16)	0.003
Pneumonia	1.29 (1.03–1.61)	0.029	1.32 (1.01–1.72)	0.045
UTI	1.01 (0.89–1.15)	0.884	1.14 (0.98–1.32)	0.086
SSI	1.50 (0.87–2.57)	0.138	1.13 (0.64–2.01)	0.672
ICU admission	1.31 (1.06–1.63)	0.014	1.38 (1.11–1.73)	0.004
RDW > 14.5%	6.57 (6.29–6.86)	<0.001	5.92 (5.63–6.23)	<0.001
Ferritin <30 ng/mL	1.52 (1.40–1.64)	<0.001	1.55 (1.42–1.68)	<0.001
CRP ≥ 10 mg/L	1.25 (1.08–1.44)	0.002	1.37 (1.13–1.66)	0.001

CI, confidence interval; HR, hazard ratio; RDW, red cell distribution width; UTI, urinary tract infection; SSI, surgical site infection; ICU, intensive care unit; CRP, C-reactive protein. For each sensitivity analysis, propensity score matching was repeated within the restricted cohort before Cox proportional hazards analysis. The contemporary ICD-10 era analysis included 10,668 matched patients per group, and the 1-week preoperative RDW analysis included 8,051 matched patients per group.

## 4 Discussion

In this large propensity score–matched cohort study, elevated preoperative RDW was associated with an increased 1-year risk of sepsis, pneumonia, and ICU admission following MBS. To our knowledge, this is the first study to examine the relationship between preoperative RDW and postoperative infectious complications in the bariatric population. Two additional findings are worth highlighting here. First, the timing of risk varied according to infection type: the association with sepsis became stronger over time, whereas the association with pneumonia diminished. Second, the association between RDW and pneumonia remained evident even among patients without baseline anemia, indicating that RDW may capture prognostic information beyond coexisting anemia.

Several pathophysiological mechanisms may underlie this association. RDW elevation has been linked to chronic inflammation and oxidative stress and may also reflect inflammation-related disturbances in iron metabolism; these interconnected processes can influence immune regulation and host defense (19–22). Inflammatory cytokines suppress erythropoietin responsiveness and disrupt iron homeostasis, leading to the release of heterogeneously sized erythrocytes, which manifest as elevated RDW (23–25). Patients undergoing MBS may be particularly susceptible to these processes because severe obesity is characterized by chronic low-grade inflammation, which can disturb iron homeostasis through hepcidin-mediated pathways, and because iron deficiency is common even before or after MBS (26–29). Our biomarker findings support these primary results. Despite balanced baseline ferritin and CRP levels after matching, elevated preoperative RDW was associated with a higher risk of postoperative iron depletion, elevated CRP, and persistent RDW elevation during follow-up. These patterns suggest that RDW may identify sustained erythropoietic and inflammatory vulnerabilities that are not fully captured by baseline laboratory values. Such vulnerability may become more evident after nutritional and metabolic stress caused by MBS. The persistent RDW elevation also suggests that the preoperative signal reflects an ongoing biological state rather than transient laboratory abnormality.

The divergent temporal profiles observed for sepsis and pneumonia are noteworthy in this study. The association with pneumonia was strongest during the early postoperative period and attenuated thereafter, possibly reflecting the convergence of anesthesia-related atelectasis, impaired respiratory mechanics in patients with obesity, and transient postoperative immune vulnerability (30–33). These perioperative factors may act in concert with the inflammatory and oxidative stress states reflected by elevated RDW, potentially contributing to an increased risk of early pulmonary infection. In contrast, the association with sepsis was not evident in the first month but strengthened during follow-up, particularly between 6 and 12 months. This delayed pattern may reflect evolving postoperative nutritional and metabolic disturbances, including iron depletion and micronutrient deficiencies, which are common after MBS and may gradually alter host defense and inflammatory regulation (34–37). Together, these findings suggest that preoperative RDW may capture a dynamic vulnerability that manifests differently across postoperative phases, with early risk driven mainly by perioperative pulmonary factors and later risk shaped by ongoing nutritional and inflammatory perturbations.

The sensitivity analysis stratified by baseline anemia status revealed a pattern that further informed the interpretation of the primary findings. Among patients with anemia, the association between elevated RDW and sepsis was stronger (HR 1.79) than that observed in the overall cohort (HR 1.42), suggesting that the coexistence of erythropoietic stress and overt hematologic impairment may be associated with a greater susceptibility to severe infection. Among patients without anemia, the association with pneumonia remained statistically significant (HR 1.33), and the point estimate for sepsis was directionally consistent, although attenuated (HR 1.26; *p* = 0.094). This pattern suggests that an elevated RDW may reflect subclinical disturbances, such as early iron depletion or inflammation-related suppression of erythropoiesis, that precede overt anemia but retain prognostic relevance. Taken together, these findings suggest that the prognostic value of RDW extends across the anemia spectrum, with the strongest associations observed when RDW elevation coexists with anemia, while a measurable signal remains even in patients without it.

Our findings are broadly consistent with those of prior studies in other surgical populations. Van Koeverden et al. reported that elevated preoperative RDW predicted postoperative sepsis and pneumonia after coronary artery bypass grafting or open abdominal aortic aneurysm repair and was associated with increased hematopoietic tissue activity, supporting a link between RDW and systemic inflammatory vulnerability (8). Elevated preoperative RDW has also been associated with increased postoperative mortality in noncardiac surgical populations, including both short- and long-term mortality outcomes (9–12). Our study extends these observations to the bariatric population, in whom obesity-related inflammation and surgery-related nutritional perturbations may create a distinct biological environment. The lack of effect modification by procedure type for the primary outcome further suggests that the prognostic relevance of preoperative RDW may apply across major bariatric techniques rather than being limited to a specific procedure.

Several limitations should be acknowledged. First, this was an observational study, and causality cannot be inferred; the E-value of 2.19 for the point estimate suggests moderate robustness to unmeasured confounding but does not exclude it. Although propensity score matching balanced available structured covariates, important unmeasured bariatric factors, including frailty, functional status, socioeconomic status, sleep apnea severity, CPAP adherence, detailed smoking exposure, perioperative care pathways, postoperative nutritional management, and center-level practice variation, were not fully captured in TriNetX. Residual confounding from these factors may therefore remain, and the findings should be interpreted as observational risk-stratification signals rather than causal evidence. Second, although multiple RDW measurements were not required for inclusion, excluding patients with discordant repeated values may have introduced selection bias if repeated preoperative testing reflected greater illness burden or more intensive evaluation. This concordant-value approach was used to reduce exposure misclassification and preserve mutually exclusive RDW exposure groups. To mitigate this concern, we performed a sensitivity analysis restricted to patients with RDW measured within 1 week before surgery, which yielded consistent results; however, individual-level RDW measurement frequency could not be directly quantified or adjusted for in TriNetX. Outcomes were ascertained through ICD-based diagnostic codes, which may introduce misclassification bias, and death ascertainment in TriNetX relies on the presence of a recorded death fact in the source electronic health record, which is known to be incomplete in some cases. In addition, ICU admission was based on structured critical care service records, and the indication for ICU-level care could not be determined; therefore, planned postoperative monitoring could not be separated from unplanned ICU admission. Third, exposure was defined using a binary RDW threshold, and the absence of a dose–response analysis limited the strength of causal inference. In addition, although TriNetX maps laboratory results to standardized concepts and units, analyzer-specific calibration and local laboratory reference ranges across sites could not be directly audited; residual inter-site laboratory heterogeneity cannot be fully excluded. Fourth, data on surgical technique, perioperative antibiotic protocols, and postoperative nutritional supplementation were not available. Fifth, although the absolute risk difference for sepsis was modest (0.35%), RDW is a routinely available and low-cost parameter, which supports its potential role in risk stratification pending formal cost-effectiveness evaluation. Finally, multiple imputation and complete-case sensitivity analyses across all laboratory variables were not feasible in the TriNetX aggregate analytic environment and could have introduced additional selection bias by restricting the cohort to patients with more intensive laboratory testing. Because TriNetX does not support patient-level longitudinal laboratory export or custom spline-based modeling, we could not evaluate alternative RDW summaries or nonlinear dose–response associations using restricted cubic splines.

## 5 Conclusion

Elevated preoperative RDW was associated with a higher 1-year risk of sepsis and pneumonia after MBS. These associations were not fully explained by baseline anemia and showed distinct temporal patterns, with the risk of pneumonia appearing earlier and the risk of sepsis becoming more evident later during follow-up. As a routinely available and low-cost hematologic marker, RDW may help identify patients with underlying inflammatory, nutritional, or erythropoietic vulnerabilities before surgery. Prospective studies are warranted to validate these findings and determine whether targeted preoperative optimization of iron status, inflammation, or other contributors to RDW elevation can reduce postoperative infectious complications.

## Data Availability

The raw data supporting the conclusions of this article will be made available by the authors, without undue reservation.
