# Supplementary material for: Elevated preoperative red cell distribution width and incident infection after metabolic and bariatric surgery: a cohort study
Source: Front Nutr. 2026 Jul 27;13:1871866. doi: 10.3389/fnut.2026.1871866 (PMC13453701; doi:10.3389/fnut.2026.1871866)
Supplement: Supplementary file 1 [file Table_1.docx]

Supplementary Material 1. TriNetX Query Definitions and Reproducibility Materials

# Methods

The analysis process includes two main steps: 1) Defining the cohorts through query criteria; 2) Setting up and running the analysis. Setting up the analysis requires definitions for the index event, outcomes criteria, and the time frame. Compare outcomes supports four analyses: Measures of Association, Survival, Number of Instances and Lab result distribution. These analyses have additional options that are listed in the Outcomes Definitions and Analyses Specifications section below. Furthermore, characteristics of the cohorts that are balanced using propensity score matching are also included in the Propensity Score Matching section.

## Cohorts definition

This section lists all terms used in the definitions of the two cohorts.

### Query Criteria for Cohort 1 (query name: ## RDW v2)

This query was run on the network Global Collaborative Network with 173 HCO(s) queried and 173 HCO(s) responded. A total of 51 provider(s) responded with patients. The final cohort included 21,337 patients who matched the query criteria listed in the table below. For the text representation of the query criteria please see Appendix A.

| Ungrouped terms | | | | | |
| --- | --- | --- | --- | --- | --- |
|  | must have |  | demographics | Age | Age (at least 18 years (most recent occurrence)) |
| Group 1 | | | | | |
|  | **Group 1A Primary Sleeve (and) RYGB** | | | | |
|  | must have | any of | procedure | UMLS:CPT:43644 | Laparoscopy, surgical, gastric restrictive procedure; with gastric bypass and Roux-en-Y gastroenterostomy (roux limb 150 cm or less) (at least 18 years old at event) |
|  |  |  | procedure | UMLS:CPT:43775 | Laparoscopy, surgical, gastric restrictive procedure; longitudinal gastrectomy (ie, sleeve gastrectomy) (at least 18 years old at event) |
|  | date constraint | | The terms in this group occurred between Jan 1, 2010 and Dec 31, 2025 | | |
|  | event relationship | | Any instance of ZD occurred within 3 months on or before any instance of Primary Sleeve (and) RYGB | | |
|  | **Group 1B ZD** | | | | |
|  | must have |  | laboratory | TNX:9008 | Erythrocyte distribution width [Ratio] (at least 14.60 %) |
|  | cannot have |  | laboratory | TNX:9008 | Erythrocyte distribution width [Ratio] (between 11.50 and 14.50 %) |
| Group 2 | | | | | |
|  | **Primary Sleeve (and) RYGB** | | | | |
|  | must have | any of | procedure | UMLS:CPT:43644 | Laparoscopy, surgical, gastric restrictive procedure; with gastric bypass and Roux-en-Y gastroenterostomy (roux limb 150 cm or less) (at least 18 years old at event) |
|  |  |  | procedure | UMLS:CPT:43775 | Laparoscopy, surgical, gastric restrictive procedure; longitudinal gastrectomy (ie, sleeve gastrectomy) (at least 18 years old at event) |
|  | date constraint | | The terms in this group occurred between Jan 1, 2010 and Dec 31, 2025 | | |
| Group 3 | | | | | |
|  | **Group 3A Primary Sleeve (and) RYGB** | | | | |
|  | must have | any of | procedure | UMLS:CPT:43644 | Laparoscopy, surgical, gastric restrictive procedure; with gastric bypass and Roux-en-Y gastroenterostomy (roux limb 150 cm or less) (at least 18 years old at event) |
|  |  |  | procedure | UMLS:CPT:43775 | Laparoscopy, surgical, gastric restrictive procedure; longitudinal gastrectomy (ie, sleeve gastrectomy) (at least 18 years old at event) |
|  | date constraint | | The terms in this group occurred between Jan 1, 2010 and Dec 31, 2025 | | |
|  | event relationship | | Any instance of Group 3B occurred at least 1 day before any instance of Primary Sleeve (and) RYGB | | |
|  | **Group 3B** | | | | |
|  | cannot have |  | diagnosis | UMLS:ICD10CM:N18.6 | End stage renal disease |
|  |  | or | diagnosis | UMLS:ICD10CM:N18.5 | Chronic kidney disease, stage 5 |
|  |  | or | diagnosis | UMLS:ICD10CM:N18.4 | Chronic kidney disease, stage 4 (severe) |
|  |  | or | diagnosis | UMLS:ICD10CM:Z99.2 | Dependence on renal dialysis |
|  |  | or | globaloncology | NAACCR:BIOM:3859 | HIV Status |
|  |  | or | diagnosis | UMLS:ICD10CM:B20 | Human immunodeficiency virus [HIV] disease |
| Group 4 | | | | | |
|  | **Group 4A Primary Sleeve (and) RYGB** | | | | |
|  | must have | any of | procedure | UMLS:CPT:43644 | Laparoscopy, surgical, gastric restrictive procedure; with gastric bypass and Roux-en-Y gastroenterostomy (roux limb 150 cm or less) (at least 18 years old at event) |
|  |  |  | procedure | UMLS:CPT:43775 | Laparoscopy, surgical, gastric restrictive procedure; longitudinal gastrectomy (ie, sleeve gastrectomy) (at least 18 years old at event) |
|  | date constraint | | The terms in this group occurred between Apr 1, 2010 and Dec 31, 2025 | | |
|  | event relationship | | Any instance of Group 4B occurred within 1 month and 1 day before any instance of Primary Sleeve (and) RYGB | | |
|  | **Group 4B** | | | | |
|  | cannot have |  | diagnosis | UMLS:ICD10CM:R65.2 | Severe sepsis |
|  |  | or | diagnosis | UMLS:ICD10CM:A41 | Other sepsis |
|  |  | or | procedure | UMLS:CPT:1013729 | Critical Care Services |
|  |  | or | diagnosis | UMLS:ICD10CM:U07.1 | COVID-19 |
|  |  | or | diagnosis | UMLS:ICD10CM:N17 | Acute kidney failure |
|  |  | or | diagnosis | UMLS:ICD10CM:J09-J18 | Influenza and pneumonia |
| Group 5 | | | | | |
|  | **Primary Sleeve (and) RYGB** | | | | |
|  | must have | any of | procedure | UMLS:CPT:43644 | Laparoscopy, surgical, gastric restrictive procedure; with gastric bypass and Roux-en-Y gastroenterostomy (roux limb 150 cm or less) (at least 18 years old at event) |
|  |  |  | procedure | UMLS:CPT:43775 | Laparoscopy, surgical, gastric restrictive procedure; longitudinal gastrectomy (ie, sleeve gastrectomy) (at least 18 years old at event) |
|  | date constraint | | The terms in this group occurred between Jan 1, 2010 and Dec 31, 2025 | | |
| Group 6 | | | | | |
|  | **Group 6A Primary Sleeve (and) RYGB** | | | | |
|  | must have | any of | procedure | UMLS:CPT:43644 | Laparoscopy, surgical, gastric restrictive procedure; with gastric bypass and Roux-en-Y gastroenterostomy (roux limb 150 cm or less) (at least 18 years old at event) |
|  |  |  | procedure | UMLS:CPT:43775 | Laparoscopy, surgical, gastric restrictive procedure; longitudinal gastrectomy (ie, sleeve gastrectomy) (at least 18 years old at event) |
|  | date constraint | | The terms in this group occurred at any time | | |
|  | event relationship | | Any instance of Group 6B occurred on the same date as any instance of Primary Sleeve (and) RYGB | | |
|  | **Group 6B** | | | | |
|  | cannot have |  | procedure | UMLS:CPT:36430 | Transfusion, blood or blood components |

### Query Criteria for Cohort 2 (query name: ## control v2)

This query was run on the network Global Collaborative Network with 173 HCO(s) queried and 173 HCO(s) responded. A total of 52 provider(s) responded with patients. The final cohort included 58,609 patients who matched the query criteria listed in the table below.

| Ungrouped terms | | | | | |
| --- | --- | --- | --- | --- | --- |
|  | must have |  | demographics | Age | Age (at least 18 years (most recent occurrence)) |
| Group 1 | | | | | |
|  | **Group 1A Primary Sleeve (and) RYGB** | | | | |
|  | must have | any of | procedure | UMLS:CPT:43644 | Laparoscopy, surgical, gastric restrictive procedure; with gastric bypass and Roux-en-Y gastroenterostomy (roux limb 150 cm or less) (at least 18 years old at event) |
|  |  |  | procedure | UMLS:CPT:43775 | Laparoscopy, surgical, gastric restrictive procedure; longitudinal gastrectomy (ie, sleeve gastrectomy) (at least 18 years old at event) |
|  | date constraint | | The terms in this group occurred between Jan 1, 2010 and Dec 31, 2025 | | |
|  | event relationship | | Any instance of ZD occurred within 3 months on or before any instance of Primary Sleeve (and) RYGB | | |
|  | **Group 1B ZD** | | | | |
|  | must have |  | laboratory | TNX:9008 | Erythrocyte distribution width [Ratio] (between 11.50 and 14.50 %) |
|  | cannot have |  | laboratory | TNX:9008 | Erythrocyte distribution width [Ratio] (at least 14.60 %) |
| Group 2 | | | | | |
|  | **Primary Sleeve (and) RYGB** | | | | |
|  | must have | any of | procedure | UMLS:CPT:43644 | Laparoscopy, surgical, gastric restrictive procedure; with gastric bypass and Roux-en-Y gastroenterostomy (roux limb 150 cm or less) (at least 18 years old at event) |
|  |  |  | procedure | UMLS:CPT:43775 | Laparoscopy, surgical, gastric restrictive procedure; longitudinal gastrectomy (ie, sleeve gastrectomy) (at least 18 years old at event) |
|  | date constraint | | The terms in this group occurred between Jan 1, 2010 and Dec 31, 2025 | | |
| Group 3 | | | | | |
|  | **Group 3A Primary Sleeve (and) RYGB** | | | | |
|  | must have | any of | procedure | UMLS:CPT:43644 | Laparoscopy, surgical, gastric restrictive procedure; with gastric bypass and Roux-en-Y gastroenterostomy (roux limb 150 cm or less) (at least 18 years old at event) |
|  |  |  | procedure | UMLS:CPT:43775 | Laparoscopy, surgical, gastric restrictive procedure; longitudinal gastrectomy (ie, sleeve gastrectomy) (at least 18 years old at event) |
|  | date constraint | | The terms in this group occurred between Jan 1, 2010 and Dec 31, 2025 | | |
|  | event relationship | | Any instance of Group 3B occurred at least 1 day before any instance of Primary Sleeve (and) RYGB | | |
|  | **Group 3B** | | | | |
|  | cannot have |  | diagnosis | UMLS:ICD10CM:N18.6 | End stage renal disease |
|  |  | or | diagnosis | UMLS:ICD10CM:N18.5 | Chronic kidney disease, stage 5 |
|  |  | or | diagnosis | UMLS:ICD10CM:N18.4 | Chronic kidney disease, stage 4 (severe) |
|  |  | or | diagnosis | UMLS:ICD10CM:Z99.2 | Dependence on renal dialysis |
|  |  | or | globaloncology | NAACCR:BIOM:3859 | HIV Status |
|  |  | or | diagnosis | UMLS:ICD10CM:B20 | Human immunodeficiency virus [HIV] disease |
| Group 4 | | | | | |
|  | **Group 4A Primary Sleeve (and) RYGB** | | | | |
|  | must have | any of | procedure | UMLS:CPT:43644 | Laparoscopy, surgical, gastric restrictive procedure; with gastric bypass and Roux-en-Y gastroenterostomy (roux limb 150 cm or less) (at least 18 years old at event) |
|  |  |  | procedure | UMLS:CPT:43775 | Laparoscopy, surgical, gastric restrictive procedure; longitudinal gastrectomy (ie, sleeve gastrectomy) (at least 18 years old at event) |
|  | date constraint | | The terms in this group occurred between Apr 1, 2010 and Dec 31, 2025 | | |
|  | event relationship | | Any instance of Group 4B occurred within 1 month and 1 day before any instance of Primary Sleeve (and) RYGB | | |
|  | **Group 4B** | | | | |
|  | cannot have |  | diagnosis | UMLS:ICD10CM:R65.2 | Severe sepsis |
|  |  | or | diagnosis | UMLS:ICD10CM:A41 | Other sepsis |
|  |  | or | procedure | UMLS:CPT:1013729 | Critical Care Services |
|  |  | or | diagnosis | UMLS:ICD10CM:U07.1 | COVID-19 |
|  |  | or | diagnosis | UMLS:ICD10CM:N17 | Acute kidney failure |
|  |  | or | diagnosis | UMLS:ICD10CM:J09-J18 | Influenza and pneumonia |
| Group 5 | | | | | |
|  | **Primary Sleeve (and) RYGB** | | | | |
|  | must have | any of | procedure | UMLS:CPT:43644 | Laparoscopy, surgical, gastric restrictive procedure; with gastric bypass and Roux-en-Y gastroenterostomy (roux limb 150 cm or less) (at least 18 years old at event) |
|  |  |  | procedure | UMLS:CPT:43775 | Laparoscopy, surgical, gastric restrictive procedure; longitudinal gastrectomy (ie, sleeve gastrectomy) (at least 18 years old at event) |
|  | date constraint | | The terms in this group occurred between Jan 1, 2010 and Dec 31, 2025 | | |
| Group 6 | | | | | |
|  | **Group 6A Primary Sleeve (and) RYGB** | | | | |
|  | must have | any of | procedure | UMLS:CPT:43644 | Laparoscopy, surgical, gastric restrictive procedure; with gastric bypass and Roux-en-Y gastroenterostomy (roux limb 150 cm or less) (at least 18 years old at event) |
|  |  |  | procedure | UMLS:CPT:43775 | Laparoscopy, surgical, gastric restrictive procedure; longitudinal gastrectomy (ie, sleeve gastrectomy) (at least 18 years old at event) |
|  | date constraint | | The terms in this group occurred at any time | | |
|  | event relationship | | Any instance of Group 6B occurred on the same date as any instance of Primary Sleeve (and) RYGB | | |
|  | **Group 6B** | | | | |
|  | cannot have |  | procedure | UMLS:CPT:36430 | Transfusion, blood or blood components |

## Analysis Setup

This section contains the Index Event and Time Window definitions and a list of selected outcomes and the analyses.

### Index Event & Time Window Definitions

The index event defines the point in time when each patient in the cohort enters the analysis. To define an index event for the cohort, one or more criteria for the cohort must be selected. The index date for each patient within a cohort is the day on which the patient first met the selected criteria for the cohort (listed in the table below).

As the index event defines the earliest time point after which outcomes are analyzed, the time window defines the duration during which outcomes are analyzed. The time window can start on the same day as the index event or at any specified time interval after the index event. The time window can end any time after the start date. Outcomes are defined as diagnoses, medications, procedures, or laboratory values that happened in the time window starting after the first occurrence of the index event.

### Time Window Used in this Analysis

This analysis included outcomes that occurred in the time window that started 1 day after the first occurrence of the index event and ended 365 days after the first occurrence of the index event.

The index event only includes events that occurred up to 20 years ago. Patients whose index event occurred 20 years or more ago are excluded. In this analysis, 0 patients in Cohort 1 and 0 patients in Cohort 2 were excluded because they met the index event more than 20 years ago.

### Index Events Used in this Analysis

Index events for the Compare Outcomes analysis were derived from the cohort definitions. Index events were defined separately for each cohort and were based on the criteria used in the original cohort definition. Please see Appendix B for the text representation of the index event definition.

The index event for Cohort 1 (query name: ## RDW v2) was defined as the following:

| Group 1 | | | | | |
| --- | --- | --- | --- | --- | --- |
|  | **Group 1A Primary Sleeve (and) RYGB** | | | | |
|  | must have | any of | procedure | UMLS:CPT:43644 | Laparoscopy, surgical, gastric restrictive procedure; with gastric bypass and Roux-en-Y gastroenterostomy (roux limb 150 cm or less) (at least 18 years old at event) |
|  |  |  | procedure | UMLS:CPT:43775 | Laparoscopy, surgical, gastric restrictive procedure; longitudinal gastrectomy (ie, sleeve gastrectomy) (at least 18 years old at event) |
|  | date constraint | | The terms in this group occurred between Jan 1, 2010 and Dec 31, 2025 | | |
|  | event relationship | | Any instance of ZD occurred within 3 months on or before any instance of Primary Sleeve (and) RYGB | | |
|  | **Group 1B ZD** | | | | |
|  | must have |  | laboratory | TNX:9008 | Erythrocyte distribution width [Ratio] (at least 14.60 %) |
|  | cannot have |  | laboratory | TNX:9008 | Erythrocyte distribution width [Ratio] (between 11.50 and 14.50 %) |

The index event for Cohort 2 (query name: ## control v2) was defined as the following:

| Group 1 | | | | | |
| --- | --- | --- | --- | --- | --- |
|  | **Group 1A Primary Sleeve (and) RYGB** | | | | |
|  | must have | any of | procedure | UMLS:CPT:43644 | Laparoscopy, surgical, gastric restrictive procedure; with gastric bypass and Roux-en-Y gastroenterostomy (roux limb 150 cm or less) (at least 18 years old at event) |
|  |  |  | procedure | UMLS:CPT:43775 | Laparoscopy, surgical, gastric restrictive procedure; longitudinal gastrectomy (ie, sleeve gastrectomy) (at least 18 years old at event) |
|  | date constraint | | The terms in this group occurred between Jan 1, 2010 and Dec 31, 2025 | | |
|  | event relationship | | Any instance of ZD occurred within 3 months on or before any instance of Primary Sleeve (and) RYGB | | |
|  | **Group 1B ZD** | | | | |
|  | must have |  | laboratory | TNX:9008 | Erythrocyte distribution width [Ratio] (between 11.50 and 14.50 %) |
|  | cannot have |  | laboratory | TNX:9008 | Erythrocyte distribution width [Ratio] (at least 14.60 %) |

### Analyses Specifications

The Compare Outcomes Analytic supports four types of analyses: Measure of Association, Survival, Number of Instances, and Lab result distribution. The first three analyses support the “exclude patients with outcomes prior to the window” setting. This option can exclude patients from the analysis if they are not at risk for an outcome (e.g., if the outcome is a chronic disease). When "exclude patients with the outcome prior to the time window" is not checked, all patients in the cohort are included in the analysis, regardless of whether they had the outcome prior to the time window. When "exclude patients with the outcome prior to the time window" is checked, patients are excluded from the analysis if their record includes the outcome prior to the beginning of the time window. This selection will exclude all patients with the outcome prior to the index event. If the start of the time window for the analysis falls some days after the index event, patients will also be excluded if they have the outcome between the index event and the start of the time window.

### Measure of Association Analysis

The Measure of Association Analysis calculates and compares the fraction of patients with the selected outcome. The output summary includes: Patients in each Cohort (count of patients meeting query criteria); Patients with Outcome in each Cohort (of the patients in the cohort, count of patients that had the outcome in the time window); and Risk (the fraction of patients in the cohort that have the outcome in the time window, i.e. Patients with Outcome / Patients in Cohort). In addition, Risk Difference (the difference in the risks in Cohort 1 and Cohort 2), Risk Ratio (the ratio of the risks in Cohort 1 and Cohort 2), and Odds Ratio (the ratio of the odds in Cohort 1 and Cohort 2). The bar chart shows the risk of the outcome for the both cohorts.

### Survival Analysis

The Kaplan-Meier Analysis estimates probability of the outcome at a respective time interval (daily time interval is used in this analysis). In order to account for the patients who exited the cohort during the analysis period, and therefore should not be included in the analysis, censoring is applied. In this analysis, patients are removed from the analysis (censored) after the last fact in their record.

The output summary includes: Patients in each Cohort (count of patients meeting query criteria); Patients with Outcome (of the patients in the cohort, count of patients that had the outcome in the time window); Median Survival (the number of days when the survival drops below 50%; the “-” indicates that survival does not drop below 50% during the time window); and Survival Probability at End of Time Window (the % survival at the end of the time window). In addition, Log-Rank test, Hazard Ratio and test for Proportionality.

### Number of Instances Analysis

The Number of Instances Analysis calculates how many times the outcome occurred in the time window. This analysis includes two additional settings: include patients with zero instances; the definition of an instance.

Selecting to exclude patients with zero instances will remove these patients from the calculations for mean number of instances, standard deviation, or median. The histogram showing the distribution of patients by number of instances will not contain a bar for zero. Alternatively, by selecting to include patients with zero instances, the mean, standard deviation, and median for number of instances will reflect these patients. The histogram will contain a bar for zero patients.

The definition of an instance affects how counts are analyzed. By selecting Date, each calendar date on which any of the terms selected in the outcome are recorded will represent one instance. For example, if the outcome is “Med A or Med B,” and a patient has “Med A” on January 3, then both medications on January 4, then “Med B” on January 6, then that patient is considered to have three instances– January 3, January 4, and January 6. Note that if an outcome occurs across several dates (e.g. Visit: inpatient encounter), then only the start date is tracked for the purpose of counting instances. A patient who begins at stay on January 1, ends that stay on January 3, begins another stay on January 10, and ends that stay on January 15, is considered to have two instances of the outcome.

Selecting Visit as an Instance will count any visit that includes the outcome as one instance, regardless of how many times it occurred. For instance, consider a patient administered an analgesic on each of the three days that make up an inpatient stay following some index event. If analgesic is an outcome, these three administrations will represent only one instance, because all three are associated with the same visit.

The output summary includes: Patients in Cohort (count of patients meeting query criteria); Patients with Outcome (of the patients in the cohort, count of patients that had the outcome in the time window); Mean (mean of the counts); Standard Deviation (standard deviation of the counts); Median (median of the counts); and Median (1+ instances) when patients with zero instances included in the analysis. In addition, T-Test statistics testing for the difference between the cohorts is included.

### Laboratory Results Analysis

Lab Results can be included in the analysis only for the outcomes that are labs. Only the most recent lab values in the time window are included. For the lab results that are numeric, the outcome summary includes: Patients in Cohort (count of patients meeting query criteria); Patients with Outcome (of the patients in the cohort, count of patients that had the outcome in the time window); Mean (mean of the counts); and Standard Deviation (the standard deviation for lab values across patients in the cohort). In addition, T-Test statistics testing for the difference between the cohorts is included.

For the non-numeric lab results, three values are reported: counts of Negative; Positives; and Unknowns.

The counts are represented in the bar chart as percentages of the total counts.

### Outcome Definitions

Table below outlines the definitions for each outcome and the analysis specifications. For outcome definitions consisting of more than one term, at least one term must match. Please see Appendix C for the text representation of the outcome definitions.

| Hb<12 | | | | |
| --- | --- | --- | --- | --- |
|  | **Outcome definition** | | | |
|  | | Laboratory | TNX:9014 | Hemoglobin [Mass/volume] in Blood (at most 12.90 g/dL (most recent occurrence)) |
|  | **Settings for the performed analyses** | | | |
|  | | Risk analysis | | including patients with outcome prior to the time window |
|  | | Kaplan - Meier survival analysis | | including patients with outcome prior to the time window |
| ICU | | | | |
|  | **Outcome definition** | | | |
|  | | Procedure | UMLS:CPT:1013729 | Critical Care Services |
|  | **Settings for the performed analyses** | | | |
|  | | Risk analysis | | including patients with outcome prior to the time window |
|  | | Kaplan - Meier survival analysis | | including patients with outcome prior to the time window |
| sepsis | | | | |
|  | **Outcome definition** | | | |
|  | | Diagnosis | UMLS:ICD10CM:R65.2 | Severe sepsis |
|  | | Diagnosis | UMLS:ICD10CM:A41 | Other sepsis |
|  | | Diagnosis | UMLS:ICD10CM:A40 | Streptococcal sepsis |
|  | **Settings for the performed analyses** | | | |
|  | | Risk analysis | | including patients with outcome prior to the time window |
|  | | Kaplan - Meier survival analysis | | including patients with outcome prior to the time window |
| RDW>14.5 | | | | |
|  | **Outcome definition** | | | |
|  | | Laboratory | TNX:9008 | Erythrocyte distribution width [Ratio] (at least 14.60 % (most recent occurrence)) |
|  | **Settings for the performed analyses** | | | |
|  | | Risk analysis | | including patients with outcome prior to the time window |
|  | | Kaplan - Meier survival analysis | | including patients with outcome prior to the time window |
| pneumonia | | | | |
|  | **Outcome definition** | | | |
|  | | Diagnosis | UMLS:ICD10CM:J12 | Viral pneumonia, not elsewhere classified |
|  | | Diagnosis | UMLS:ICD10CM:J13 | Pneumonia due to Streptococcus pneumoniae |
|  | | Diagnosis | UMLS:ICD10CM:J14 | Pneumonia due to Hemophilus influenzae |
|  | | Diagnosis | UMLS:ICD10CM:J15 | Bacterial pneumonia, not elsewhere classified |
|  | | Diagnosis | UMLS:ICD10CM:J16 | Pneumonia due to other infectious organisms, not elsewhere classified |
|  | | Diagnosis | UMLS:ICD10CM:J17 | Pneumonia in diseases classified elsewhere |
|  | | Diagnosis | UMLS:ICD10CM:J18 | Pneumonia, unspecified organism |
|  | | Diagnosis | UMLS:ICD9CM:483 | Pneumonia due to other specified organism |
|  | **Settings for the performed analyses** | | | |
|  | | Risk analysis | | including patients with outcome prior to the time window |
|  | | Kaplan - Meier survival analysis | | including patients with outcome prior to the time window |
| SSI | | | | |
|  | **Outcome definition** | | | |
|  | | Diagnosis | UMLS:ICD10CM:T81.41 | Infection following a procedure, superficial incisional surgical site |
|  | | Diagnosis | UMLS:ICD10CM:T81.42 | Infection following a procedure, deep incisional surgical site |
|  | **Settings for the performed analyses** | | | |
|  | | Kaplan - Meier survival analysis | | including patients with outcome prior to the time window |
|  | | Risk analysis | | including patients with outcome prior to the time window |
| Ferritin<30 | | | | |
|  | **Outcome definition** | | | |
|  | | Laboratory | TNX:9042 | Ferritin [Mass/volume] in Serum, Plasma or Blood (at most 29.90 ng/mL (most recent occurrence)) |
|  | **Settings for the performed analyses** | | | |
|  | | Kaplan - Meier survival analysis | | including patients with outcome prior to the time window |
|  | | Risk analysis | | including patients with outcome prior to the time window |
| urinary tract infection | | | | |
|  | **Outcome definition** | | | |
|  | | Diagnosis | UMLS:ICD10CM:N39.0 | Urinary tract infection, site not specified |
|  | **Settings for the performed analyses** | | | |
|  | | Risk analysis | | including patients with outcome prior to the time window |
|  | | Kaplan - Meier survival analysis | | including patients with outcome prior to the time window |
| CRP | | | | |
|  | **Outcome definition** | | | |
|  | | Laboratory | TNX:9063 | C reactive protein [Mass/volume] in Serum, Plasma or Blood (at least 10.00 mg/L (most recent occurrence)) |
|  | **Settings for the performed analyses** | | | |
|  | | Kaplan - Meier survival analysis | | including patients with outcome prior to the time window |
|  | | Risk analysis | | including patients with outcome prior to the time window |

## Propensity Score Matching

Propensity score matching was performed on all listed characteristics. Characteristics of the cohorts before and after matching are summarized in the table below.

| **Cohort 1 and cohort 2 patient count before and after propensity score matching** | | | | | | | | | | | | | | |
| --- | --- | --- | --- | --- | --- | --- | --- | --- | --- | --- | --- | --- | --- | --- |
|  | | | Cohort | | | Patient count before matching | | | | | | Patient count after matching | | |
|  | | | 1 - ## RDW v2 | | | 21,089 | | | | | | 17,735 | | |
|  | | | 2 - ## control v2 | | | 57,995 | | | | | | 17,735 | | |
| **Propensity score density function - Before and after matching (cohort 1 - purple, cohort 2 - green)** | | | | | | | | | | | | | | |
|  |  | |  | | | | | |  | | | | | |
| **Cohort 1 (N = 21,089) and cohort 2 (N = 57,995) characteristics before propensity score matching** | | | | | | | | | | | | | | |
|  | **Demographics** | | | | | | | | | | | | | |
|  |  | Cohort | | |  | Mean ± SD | Median | IQR | | Patients | % of Cohort | | P-Value | Std diff. |
|  |  | 1 2 | | AI | Age at Index | 43.5 +/- 11.9 43.2 +/- 11.8 | 43 43 | 18 18 | | 21,089 57,995 | 100% 100% | | 0.001 | 0.027 |
|  |  | 1 2 | | 2106-3 | White |  |  |  | | 10,262 40,150 | 48.7% 69.2% | | <0.001 | 0.428 |
|  |  | 1 2 | | F | Female |  |  |  | | 18,311 46,821 | 86.8% 80.7% | | <0.001 | 0.166 |
|  |  | 1 2 | | 2054-5 | Black or African American |  |  |  | | 8,054 10,072 | 38.2% 17.4% | | <0.001 | 0.478 |
|  |  | 1 2 | | 2028-9 | Asian |  |  |  | | 131 351 | 0.6% 0.6% | | 0.799 | 0.002 |
|  | **Diagnosis** | | | | | | | | | | | | | |
|  |  | Cohort | | |  | Mean ± SD | Median | IQR | | Patients | % of Cohort | | P-Value | Std diff. |
|  |  | 1 2 | | I10 | Essential (primary) hypertension |  |  |  | | 13,481 33,293 | 63.9% 57.4% | | <0.001 | 0.134 |
|  |  | 1 2 | | C00-D49 | Neoplasms |  |  |  | | 4,306 10,763 | 20.4% 18.6% | | <0.001 | 0.047 |
|  |  | 1 2 | | E08-E13 | Diabetes mellitus |  |  |  | | 8,093 17,642 | 38.4% 30.4% | | <0.001 | 0.168 |
|  |  | 1 2 | | F17 | Nicotine dependence |  |  |  | | 2,122 6,069 | 10.1% 10.5% | | 0.100 | 0.013 |
|  |  | 1 2 | | I20-I25 | Ischemic heart diseases |  |  |  | | 1,960 4,093 | 9.3% 7.1% | | <0.001 | 0.082 |
|  |  | 1 2 | | N18 | Chronic kidney disease (CKD) |  |  |  | | 1,032 1,852 | 4.9% 3.2% | | <0.001 | 0.086 |
|  |  | 1 2 | | I60-I69 | Cerebrovascular diseases |  |  |  | | 564 1,138 | 2.7% 2.0% | | <0.001 | 0.047 |
|  |  | 1 2 | | F10 | Alcohol related disorders |  |  |  | | 426 1,184 | 2.0% 2.0% | | 0.850 | 0.002 |
|  |  | 1 2 | | J44 | Other chronic obstructive pulmonary disease |  |  |  | | 1,079 1,974 | 5.1% 3.4% | | <0.001 | 0.085 |
|  |  | 1 2 | | K70-K77 | Diseases of liver |  |  |  | | 5,162 15,105 | 24.5% 26.0% | | <0.001 | 0.036 |
|  |  | 1 2 | | G47.3 | Sleep apnea |  |  |  | | 13,241 34,203 | 62.8% 59.0% | | <0.001 | 0.078 |
|  |  | 1 2 | | F33 | Major depressive disorder, recurrent |  |  |  | | 2,448 6,631 | 11.6% 11.4% | | 0.497 | 0.005 |
|  |  | 1 2 | | F41 | Other anxiety disorders |  |  |  | | 7,912 23,771 | 37.5% 41.0% | | <0.001 | 0.071 |
|  |  | 1 2 | | D50 | Iron deficiency anemia |  |  |  | | 4,390 3,604 | 20.8% 6.2% | | <0.001 | 0.437 |
|  |  | 1 2 | | D51 | Vitamin B12 deficiency anemia |  |  |  | | 178 401 | 0.8% 0.7% | | 0.026 | 0.017 |
|  | **Procedure** | | | | | | | | | | | | | |
|  |  | Cohort | | |  | Mean ± SD | Median | IQR | | Patients | % of Cohort | | P-Value | Std diff. |
|  |  | 1 2 | | 43644 | Laparoscopy, surgical, gastric restrictive procedure; with gastric bypass and Roux-en-Y gastroenterostomy (roux limb 150 cm or less) |  |  |  | | 6,727 20,550 | 31.9% 35.4% | | <0.001 | 0.075 |
|  |  | 1 2 | | 43775 | Laparoscopy, surgical, gastric restrictive procedure; longitudinal gastrectomy (ie, sleeve gastrectomy) |  |  |  | | 14,486 37,719 | 68.7% 65.0% | | <0.001 | 0.078 |
|  | **Medication** | | | | | | | | | | | | | |
|  |  | Cohort | | |  | Mean ± SD | Median | IQR | | Patients | % of Cohort | | P-Value | Std diff. |
|  |  | 1 2 | | A10BK | Sodium-glucose co-transporter 2 (SGLT2) inhibitors |  |  |  | | 877 1,715 | 4.2% 3.0% | | <0.001 | 0.065 |
|  |  | 1 2 | | A10A | INSULINS AND ANALOGUES |  |  |  | | 6,257 13,758 | 29.7% 23.7% | | <0.001 | 0.135 |
|  |  | 1 2 | | A10BJ | Glucagon-like peptide-1 (GLP-1) analogues |  |  |  | | 3,171 8,004 | 15.0% 13.8% | | <0.001 | 0.035 |
|  |  | 1 2 | | TN410 | IRON |  |  |  | | 4,757 5,219 | 22.6% 9.0% | | <0.001 | 0.379 |
|  |  | 1 2 | | H02 | CORTICOSTEROIDS FOR SYSTEMIC USE |  |  |  | | 16,303 44,929 | 77.3% 77.5% | | 0.624 | 0.004 |
|  | **Laboratory** | | | | | | | | | | | | | |
|  |  | Cohort | | |  | Mean ± SD | Median | IQR | | Patients | % of Cohort | | P-Value | Std diff. |
|  |  | 1 2 | | 9045 | Albumin [Mass/volume] in Serum, Plasma or Blood | 4.0 +/- 0.4 4.2 +/- 0.4 | 4 4.200 | 0.400 0.500 | | 19,358 52,757 | 91.8% 91.0% | | <0.001 | 0.439 |
|  |  | 1 2 | |  | 3.50 - 0 g/dL |  |  |  | | 18,546 51,938 | 87.9% 89.6% | | <0.001 | 0.051 |
|  |  | 1 2 | | 9037 | Hemoglobin A1c/Hemoglobin.total in Blood | 6.1 +/- 1.1 5.9 +/- 1.1 | 5.800 5.600 | 0.800 0.800 | | 17,167 45,997 | 81.4% 79.3% | | <0.001 | 0.183 |
|  |  | 1 2 | |  | 7 - 0 % |  |  |  | | 3,346 7,089 | 15.9% 12.2% | | <0.001 | 0.105 |
|  |  | 1 2 | | 9083 | BMI | 46.7 +/- 8.5 44.0 +/- 7.3 | 45.725 43 | 10.843 8.660 | | 17,678 49,652 | 83.8% 85.6% | | <0.001 | 0.346 |
|  |  | 1 2 | |  | 35 - 40 kg/m2 |  |  |  | | 5,139 19,524 | 24.4% 33.7% | | <0.001 | 0.206 |
|  |  | 1 2 | |  | 40 - 45 kg/m2 |  |  |  | | 8,292 27,672 | 39.3% 47.7% | | <0.001 | 0.170 |
|  |  | 1 2 | |  | 45 - 50 kg/m2 |  |  |  | | 7,808 20,667 | 37.0% 35.6% | | <0.001 | 0.029 |
|  |  | 1 2 | | 9014 | Hemoglobin [Mass/volume] in Blood | 12.5 +/- 1.4 13.6 +/- 1.2 | 12.400 13.600 | 1.700 1.600 | | 20,996 57,785 | 99.6% 99.6% | | <0.001 | 0.853 |
|  |  | 1 2 | |  | 12 - 0 g/dL |  |  |  | | 16,491 56,194 | 78.2% 96.9% | | <0.001 | 0.590 |
|  |  | 1 2 | | 9063 | C reactive protein [Mass/volume] in Serum, Plasma or Blood | 22.6 +/- 39.2 14.6 +/- 25.6 | 12.700 8.100 | 16.730 11.300 | | 2,934 7,140 | 13.9% 12.3% | | <0.001 | 0.242 |
|  |  | 1 2 | |  | 10 - 0 mg/L |  |  |  | | 1,887 3,410 | 8.9% 5.9% | | <0.001 | 0.117 |
|  |  | 1 2 | | 9042 | Ferritin [Mass/volume] in Serum, Plasma or Blood | 67.0 +/- 125.7 112.3 +/- 127.6 | 37 77 | 62 99 | | 11,552 30,944 | 54.8% 53.4% | | <0.001 | 0.358 |
|  |  | 1 2 | |  | 0 - 30 ng/mL |  |  |  | | 5,546 5,854 | 26.3% 10.1% | | <0.001 | 0.430 |
|  |  | 1 2 | | 9065 | Cobalamin (Vitamin B12) [Mass/volume] in Serum, Plasma or Blood | 552.8 +/- 356.1 550.1 +/- 307.4 | 477 480 | 299 284 | | 13,114 37,769 | 62.2% 65.1% | | 0.413 | 0.008 |
|  |  | 1 2 | |  | 0 - 300 pg/mL |  |  |  | | 2,268 5,856 | 10.8% 10.1% | | 0.007 | 0.022 |
|  |  | 1 2 | | 6690-2 | Leukocytes [#/volume] in Blood by Automated count | 9.1 +/- 36.9 9.1 +/- 45.1 | 8.300 8.100 | 3.660 3.500 | | 15,586 44,373 | 73.9% 76.5% | | 0.908 | 0.001 |
|  |  | 1 2 | |  | 11 - 0 10*3/uL |  |  |  | | 5,579 13,934 | 26.5% 24.0% | | <0.001 | 0.056 |
|  |  | 1 2 | | 8001 | Glomerular filtration rate/1.73 sq M.predicted [Volume Rate/Area] in Serum, Plasma or Blood by Creatinine-based formula (MDRD) | 92.6 +/- 25.8 90.9 +/- 23.2 | 91.159 89.290 | 32.075 28.593 | | 20,126 55,985 | 95.4% 96.5% | | <0.001 | 0.069 |
|  |  | 1 2 | |  | 60 - 0 mL/min/{1.73_m2} |  |  |  | | 19,501 54,701 | 92.5% 94.3% | | <0.001 | 0.075 |
| **Cohort 1 (N = 17,735) and cohort 2 (N = 17,735) characteristics after propensity score matching** | | | | | | | | | | | | | | |
|  | **Demographics** | | | | | | | | | | | | | |
|  |  | Cohort | | |  | Mean ± SD | Median | IQR | | Patients | % of Cohort | | P-Value | Std diff. |
|  |  | 1 2 | | AI | Age at Index | 43.9 +/- 11.9 44.2 +/- 11.9 | 43 44 | 18 18 | | 17,735 17,735 | 100% 100% | | 0.018 | 0.025 |
|  |  | 1 2 | | 2106-3 | White |  |  |  | | 9,528 9,403 | 53.7% 53.0% | | 0.183 | 0.014 |
|  |  | 1 2 | | F | Female |  |  |  | | 15,062 15,089 | 84.9% 85.1% | | 0.688 | 0.004 |
|  |  | 1 2 | | 2054-5 | Black or African American |  |  |  | | 5,808 5,967 | 32.7% 33.6% | | 0.073 | 0.019 |
|  |  | 1 2 | | 2028-9 | Asian |  |  |  | | 112 104 | 0.6% 0.6% | | 0.585 | 0.006 |
|  | **Diagnosis** | | | | | | | | | | | | | |
|  |  | Cohort | | |  | Mean ± SD | Median | IQR | | Patients | % of Cohort | | P-Value | Std diff. |
|  |  | 1 2 | | I10 | Essential (primary) hypertension |  |  |  | | 11,324 11,473 | 63.9% 64.7% | | 0.099 | 0.018 |
|  |  | 1 2 | | C00-D49 | Neoplasms |  |  |  | | 3,600 3,628 | 20.3% 20.5% | | 0.712 | 0.004 |
|  |  | 1 2 | | E08-E13 | Diabetes mellitus |  |  |  | | 6,760 6,770 | 38.1% 38.2% | | 0.913 | 0.001 |
|  |  | 1 2 | | F17 | Nicotine dependence |  |  |  | | 1,796 1,826 | 10.1% 10.3% | | 0.599 | 0.006 |
|  |  | 1 2 | | I20-I25 | Ischemic heart diseases |  |  |  | | 1,656 1,720 | 9.3% 9.7% | | 0.247 | 0.012 |
|  |  | 1 2 | | N18 | Chronic kidney disease (CKD) |  |  |  | | 860 843 | 4.8% 4.8% | | 0.673 | 0.004 |
|  |  | 1 2 | | I60-I69 | Cerebrovascular diseases |  |  |  | | 463 487 | 2.6% 2.7% | | 0.430 | 0.008 |
|  |  | 1 2 | | F10 | Alcohol related disorders |  |  |  | | 369 370 | 2.1% 2.1% | | 0.970 | <0.001 |
|  |  | 1 2 | | J44 | Other chronic obstructive pulmonary disease |  |  |  | | 901 946 | 5.1% 5.3% | | 0.282 | 0.011 |
|  |  | 1 2 | | K70-K77 | Diseases of liver |  |  |  | | 4,457 4,406 | 25.1% 24.8% | | 0.532 | 0.007 |
|  |  | 1 2 | | G47.3 | Sleep apnea |  |  |  | | 11,189 11,282 | 63.1% 63.6% | | 0.305 | 0.011 |
|  |  | 1 2 | | F33 | Major depressive disorder, recurrent |  |  |  | | 2,098 2,026 | 11.8% 11.4% | | 0.233 | 0.013 |
|  |  | 1 2 | | F41 | Other anxiety disorders |  |  |  | | 6,841 6,831 | 38.6% 38.5% | | 0.913 | 0.001 |
|  |  | 1 2 | | D50 | Iron deficiency anemia |  |  |  | | 2,602 2,563 | 14.7% 14.5% | | 0.557 | 0.006 |
|  |  | 1 2 | | D51 | Vitamin B12 deficiency anemia |  |  |  | | 145 159 | 0.8% 0.9% | | 0.420 | 0.009 |
|  | **Procedure** | | | | | | | | | | | | | |
|  |  | Cohort | | |  | Mean ± SD | Median | IQR | | Patients | % of Cohort | | P-Value | Std diff. |
|  |  | 1 2 | | 43644 | Laparoscopy, surgical, gastric restrictive procedure; with gastric bypass and Roux-en-Y gastroenterostomy (roux limb 150 cm or less) |  |  |  | | 5,759 5,667 | 32.5% 32.0% | | 0.296 | 0.011 |
|  |  | 1 2 | | 43775 | Laparoscopy, surgical, gastric restrictive procedure; longitudinal gastrectomy (ie, sleeve gastrectomy) |  |  |  | | 12,083 12,167 | 68.1% 68.6% | | 0.338 | 0.010 |
|  | **Medication** | | | | | | | | | | | | | |
|  |  | Cohort | | |  | Mean ± SD | Median | IQR | | Patients | % of Cohort | | P-Value | Std diff. |
|  |  | 1 2 | | A10BK | Sodium-glucose co-transporter 2 (SGLT2) inhibitors |  |  |  | | 751 748 | 4.2% 4.2% | | 0.937 | 0.001 |
|  |  | 1 2 | | A10A | INSULINS AND ANALOGUES |  |  |  | | 5,225 5,267 | 29.5% 29.7% | | 0.625 | 0.005 |
|  |  | 1 2 | | A10BJ | Glucagon-like peptide-1 (GLP-1) analogues |  |  |  | | 2,666 2,645 | 15.0% 14.9% | | 0.755 | 0.003 |
|  |  | 1 2 | | TN410 | IRON |  |  |  | | 3,096 2,998 | 17.5% 16.9% | | 0.168 | 0.015 |
|  |  | 1 2 | | H02 | CORTICOSTEROIDS FOR SYSTEMIC USE |  |  |  | | 13,706 13,765 | 77.3% 77.6% | | 0.453 | 0.008 |
|  | **Laboratory** | | | | | | | | | | | | | |
|  |  | Cohort | | |  | Mean ± SD | Median | IQR | | Patients | % of Cohort | | P-Value | Std diff. |
|  |  | 1 2 | | 9045 | Albumin [Mass/volume] in Serum, Plasma or Blood | 4.0 +/- 0.4 4.1 +/- 0.4 | 4 4.100 | 0.500 0.400 | | 16,269 16,060 | 91.7% 90.6% | | <0.001 | 0.224 |
|  |  | 1 2 | |  | 3.50 - 0 g/dL |  |  |  | | 15,679 15,673 | 88.4% 88.4% | | 0.921 | 0.001 |
|  |  | 1 2 | | 9037 | Hemoglobin A1c/Hemoglobin.total in Blood | 6.1 +/- 1.1 5.9 +/- 1.3 | 5.800 5.700 | 0.800 1 | | 14,276 14,505 | 80.5% 81.8% | | <0.001 | 0.098 |
|  |  | 1 2 | |  | 7 - 0 % |  |  |  | | 2,822 2,815 | 15.9% 15.9% | | 0.919 | 0.001 |
|  |  | 1 2 | | 9083 | BMI | 46.4 +/- 8.3 45.2 +/- 7.9 | 45.354 44.572 | 10.608 9.780 | | 14,778 14,643 | 83.3% 82.6% | | <0.001 | 0.155 |
|  |  | 1 2 | |  | 35 - 40 kg/m2 |  |  |  | | 4,555 4,421 | 25.7% 24.9% | | 0.102 | 0.017 |
|  |  | 1 2 | |  | 40 - 45 kg/m2 |  |  |  | | 7,157 6,927 | 40.4% 39.1% | | 0.013 | 0.027 |
|  |  | 1 2 | |  | 45 - 50 kg/m2 |  |  |  | | 6,514 6,450 | 36.7% 36.4% | | 0.480 | 0.007 |
|  |  | 1 2 | | 9014 | Hemoglobin [Mass/volume] in Blood | 12.7 +/- 1.4 13.3 +/- 1.3 | 12.700 13.300 | 1.600 1.700 | | 17,688 17,532 | 99.7% 98.9% | | <0.001 | 0.414 |
|  |  | 1 2 | |  | 12 - 0 g/dL |  |  |  | | 15,750 15,965 | 88.8% 90.0% | | <0.001 | 0.039 |
|  |  | 1 2 | | 9063 | C reactive protein [Mass/volume] in Serum, Plasma or Blood | 22.1 +/- 38.3 16.3 +/- 26.6 | 12.200 9.200 | 16.170 13.330 | | 2,379 2,798 | 13.4% 15.8% | | <0.001 | 0.177 |
|  |  | 1 2 | |  | 10 - 0 mg/L |  |  |  | | 1,497 1,523 | 8.4% 8.6% | | 0.621 | 0.005 |
|  |  | 1 2 | | 9042 | Ferritin [Mass/volume] in Serum, Plasma or Blood | 73.2 +/- 134.6 95.9 +/- 119.6 | 43 61 | 68.700 94.300 | | 9,130 10,433 | 51.5% 58.8% | | <0.001 | 0.178 |
|  |  | 1 2 | |  | 0 - 30 ng/mL |  |  |  | | 3,659 3,735 | 20.6% 21.1% | | 0.321 | 0.011 |
|  |  | 1 2 | | 9065 | Cobalamin (Vitamin B12) [Mass/volume] in Serum, Plasma or Blood | 554.0 +/- 365.7 560.5 +/- 335.8 | 477 484 | 300 294 | | 10,790 11,609 | 60.8% 65.5% | | 0.160 | 0.019 |
|  |  | 1 2 | |  | 0 - 300 pg/mL |  |  |  | | 1,859 1,829 | 10.5% 10.3% | | 0.602 | 0.006 |
|  |  | 1 2 | | 6690-2 | Leukocytes [#/volume] in Blood by Automated count | 8.9 +/- 3.3 9.3 +/- 58.0 | 8.340 8 | 3.670 3.700 | | 12,999 13,832 | 73.3% 78.0% | | 0.429 | 0.010 |
|  |  | 1 2 | |  | 11 - 0 10*3/uL |  |  |  | | 4,659 4,594 | 26.3% 25.9% | | 0.432 | 0.008 |
|  |  | 1 2 | | 8001 | Glomerular filtration rate/1.73 sq M.predicted [Volume Rate/Area] in Serum, Plasma or Blood by Creatinine-based formula (MDRD) | 91.6 +/- 25.4 91.0 +/- 24.9 | 90.100 89.694 | 31.494 30.968 | | 16,950 16,993 | 95.6% 95.8% | | 0.017 | 0.026 |
|  |  | 1 2 | |  | 60 - 0 mL/min/{1.73_m2} |  |  |  | | 16,432 16,447 | 92.7% 92.7% | | 0.760 | 0.003 |

# Results

Results are summarized in the tables below. Outcomes analysis was performed on the cohorts after propensity score matching.

| **Follow-up Time (Before Matching)** | | | | | | |
| --- | --- | --- | --- | --- | --- | --- |
|  |  | Cohort | Mean Follow-up (Days) | Standard Deviation | Median Follow-up (Days) | Interquartile Range |
|  |  | ## RDW v2 | 340.656 | 73.168 | 365 | 0 |
|  |  | ## control v2 | 338.856 | 75.683 | 365 | 0 |
|  | | | | | | |
|  | | | | | | |
| **Follow-up Time (After Matching)** | | | | | | |
|  |  | Cohort | Mean Follow-up (Days) | Standard Deviation | Median Follow-up (Days) | Interquartile Range |
|  |  | ## RDW v2 | 341.126 | 72.593 | 365 | 0 |
|  |  | ## control v2 | 338.424 | 75.891 | 365 | 0 |
|  | | | | | | |

| **2 ICU** | | | | | | | | | | | | |
| --- | --- | --- | --- | --- | --- | --- | --- | --- | --- | --- | --- | --- |
|  | | **Risk analysis** | | | | | | | | | | |
|  |  | | | Cohort | | | Patients in cohort | Patients with outcome | Risk | | | |
|  | | |  | 1 | | ## RDW v2 | 17,735 | 312 | 0.018 | | | |
|  | | |  | 2 | | ## control v2 | 17,735 | 255 | 0.014 | | | |
|  | | | | | | | | | | | | |
|  | | |  |  | | |  | 95% CI | z | p |  |  |
|  | | |  | **Risk Difference** | | | 0.003 | (0.001, 0.006) | 2.413 | 0.016 |  |  |
|  | | |  | **Risk Ratio** | | | 1.224 | (1.038, 1.442) | N/A | N/A |  |  |
|  | | |  | **Odds Ratio** | | | 1.228 | (1.039, 1.450) | N/A | N/A |  |  |
|  | | | | | | | | | | | | |
|  | |  | | |  | | | | | | | |
|  | | **Kaplan - Meier survival analysis** | | | | | | | | | | |
|  | | |  | Cohort | | | Patients in cohort | Patients with outcome | Median survival (days) | Survival probability at end of time window | | |
|  | | |  | 1 | | ## RDW v2 | 17,735 | 312 | -- | 98.18% | | |
|  | | |  | 2 | | ## control v2 | 17,735 | 255 | -- | 98.51% | | |
|  | | | | | | | | | | | | |
|  | | |  |  | | | χ^2^ | df | p |  |  |  |
|  | | |  | **Log-Rank Test** | | | 5.601 | 1 | 0.018 |  |  |  |
|  | | | | | | | | | | | | |
|  | | |  |  | | | Hazard Ratio | 95% CI | χ^2^ | df | p | |
|  | | |  | **Hazard Ratio and Proportionality** | | | 1.221 | (1.035, 1.440) | 1.039 | 1 | 0.308 | |
|  | | | | | | | | | | | | |
|  | |  | | |  | | | | | | | |
| **3 sepsis** | | | | | | | | | | | | |
|  | | **Risk analysis** | | | | | | | | | | |
|  |  | | | Cohort | | | Patients in cohort | Patients with outcome | Risk | | | |
|  | | |  | 1 | | ## RDW v2 | 17,735 | 209 | 0.012 | | | |
|  | | |  | 2 | | ## control v2 | 17,735 | 147 | 0.008 | | | |
|  | | | | | | | | | | | | |
|  | | |  |  | | |  | 95% CI | z | p |  |  |
|  | | |  | **Risk Difference** | | | 0.003 | (0.001, 0.006) | 3.303 | 0.001 |  |  |
|  | | |  | **Risk Ratio** | | | 1.422 | (1.153, 1.754) | N/A | N/A |  |  |
|  | | |  | **Odds Ratio** | | | 1.427 | (1.154, 1.764) | N/A | N/A |  |  |
|  | | | | | | | | | | | | |
|  | |  | | |  | | | | | | | |
|  | | **Kaplan - Meier survival analysis** | | | | | | | | | | |
|  | | |  | Cohort | | | Patients in cohort | Patients with outcome | Median survival (days) | Survival probability at end of time window | | |
|  | | |  | 1 | | ## RDW v2 | 17,735 | 209 | -- | 98.77% | | |
|  | | |  | 2 | | ## control v2 | 17,735 | 147 | -- | 99.13% | | |
|  | | | | | | | | | | | | |
|  | | |  |  | | | χ^2^ | df | p |  |  |  |
|  | | |  | **Log-Rank Test** | | | 10.600 | 1 | 0.001 |  |  |  |
|  | | | | | | | | | | | | |
|  | | |  |  | | | Hazard Ratio | 95% CI | χ^2^ | df | p | |
|  | | |  | **Hazard Ratio and Proportionality** | | | 1.417 | (1.148, 1.750) | 2.060 | 1 | 0.151 | |
|  | | | | | | | | | | | | |
|  | |  | | |  | | | | | | | |
| **4 RDW>14.5** | | | | | | | | | | | | |
|  | | **Risk analysis** | | | | | | | | | | |
|  |  | | | Cohort | | | Patients in cohort | Patients with outcome | Risk | | | |
|  | | |  | 1 | | ## RDW v2 | 17,735 | 15,045 | 0.848 | | | |
|  | | |  | 2 | | ## control v2 | 17,735 | 4,472 | 0.252 | | | |
|  | | | | | | | | | | | | |
|  | | |  |  | | |  | 95% CI | z | p |  |  |
|  | | |  | **Risk Difference** | | | 0.596 | (0.588, 0.604) | 112.850 | 0 |  |  |
|  | | |  | **Risk Ratio** | | | 3.364 | (3.278, 3.453) | N/A | N/A |  |  |
|  | | |  | **Odds Ratio** | | | 16.587 | (15.728, 17.494) | N/A | N/A |  |  |
|  | | | | | | | | | | | | |
|  | |  | | |  | | | | | | | |
|  | | **Kaplan - Meier survival analysis** | | | | | | | | | | |
|  | | |  | Cohort | | | Patients in cohort | Patients with outcome | Median survival (days) | Survival probability at end of time window | | |
|  | | |  | 1 | | ## RDW v2 | 17,735 | 15,045 | 1 | 14.50% | | |
|  | | |  | 2 | | ## control v2 | 17,735 | 4,472 | -- | 74.02% | | |
|  | | | | | | | | | | | | |
|  | | |  |  | | | χ^2^ | df | p |  |  |  |
|  | | |  | **Log-Rank Test** | | | 16448.779 | 1 | 0 |  |  |  |
|  | | | | | | | | | | | | |
|  | | |  |  | | | Hazard Ratio | 95% CI | χ^2^ | df | p | |
|  | | |  | **Hazard Ratio and Proportionality** | | | 6.532 | (6.311, 6.761) | 400.728 | 1 | 0.000 | |
|  | | | | | | | | | | | | |
|  | |  | | |  | | | | | | | |
| **5 pneumonia** | | | | | | | | | | | | |
|  | | **Risk analysis** | | | | | | | | | | |
|  |  | | | Cohort | | | Patients in cohort | Patients with outcome | Risk | | | |
|  | | |  | 1 | | ## RDW v2 | 17,735 | 267 | 0.015 | | | |
|  | | |  | 2 | | ## control v2 | 17,735 | 201 | 0.011 | | | |
|  | | | | | | | | | | | | |
|  | | |  |  | | |  | 95% CI | z | p |  |  |
|  | | |  | **Risk Difference** | | | 0.004 | (0.001, 0.006) | 3.071 | 0.002 |  |  |
|  | | |  | **Risk Ratio** | | | 1.328 | (1.107, 1.593) | N/A | N/A |  |  |
|  | | |  | **Odds Ratio** | | | 1.333 | (1.109, 1.603) | N/A | N/A |  |  |
|  | | | | | | | | | | | | |
|  | |  | | |  | | | | | | | |
|  | | **Kaplan - Meier survival analysis** | | | | | | | | | | |
|  | | |  | Cohort | | | Patients in cohort | Patients with outcome | Median survival (days) | Survival probability at end of time window | | |
|  | | |  | 1 | | ## RDW v2 | 17,735 | 267 | -- | 98.43% | | |
|  | | |  | 2 | | ## control v2 | 17,735 | 201 | -- | 98.80% | | |
|  | | | | | | | | | | | | |
|  | | |  |  | | | χ^2^ | df | p |  |  |  |
|  | | |  | **Log-Rank Test** | | | 9.149 | 1 | 0.002 |  |  |  |
|  | | | | | | | | | | | | |
|  | | |  |  | | | Hazard Ratio | 95% CI | χ^2^ | df | p | |
|  | | |  | **Hazard Ratio and Proportionality** | | | 1.325 | (1.104, 1.591) | 3.264 | 1 | 0.071 | |
|  | | | | | | | | | | | | |
|  | |  | | |  | | | | | | | |
| **6 SSI** | | | | | | | | | | | | |
|  | | **Risk analysis** | | | | | | | | | | |
|  |  | | | Cohort | | | Patients in cohort | Patients with outcome | Risk | | | |
|  | | |  | 1 | | ## RDW v2 | 17,735 | 42 | 0.002 | | | |
|  | | |  | 2 | | ## control v2 | 17,735 | 40 | 0.002 | | | |
|  | | | | | | | | | | | | |
|  | | |  |  | | |  | 95% CI | z | p |  |  |
|  | | |  | **Risk Difference** | | | 0.000 | (-0.001, 0.001) | 0.221 | 0.825 |  |  |
|  | | |  | **Risk Ratio** | | | 1.050 | (0.681, 1.618) | N/A | N/A |  |  |
|  | | |  | **Odds Ratio** | | | 1.050 | (0.681, 1.620) | N/A | N/A |  |  |
|  | | | | | | | | | | | | |
|  | |  | | |  | | | | | | | |
|  | | **Kaplan - Meier survival analysis** | | | | | | | | | | |
|  | | |  | Cohort | | | Patients in cohort | Patients with outcome | Median survival (days) | Survival probability at end of time window | | |
|  | | |  | 1 | | ## RDW v2 | 17,735 | 42 | -- | 99.76% | | |
|  | | |  | 2 | | ## control v2 | 17,735 | 40 | -- | 99.77% | | |
|  | | | | | | | | | | | | |
|  | | |  |  | | | χ^2^ | df | p |  |  |  |
|  | | |  | **Log-Rank Test** | | | 0.043 | 1 | 0.837 |  |  |  |
|  | | | | | | | | | | | | |
|  | | |  |  | | | Hazard Ratio | 95% CI | χ^2^ | df | p | |
|  | | |  | **Hazard Ratio and Proportionality** | | | 1.047 | (0.679, 1.614) | 0.078 | 1 | 0.780 | |
|  | | | | | | | | | | | | |
|  | |  | | |  | | | | | | | |
| **7 Ferritin<30** | | | | | | | | | | | | |
|  | | **Risk analysis** | | | | | | | | | | |
|  |  | | | Cohort | | | Patients in cohort | Patients with outcome | Risk | | | |
|  | | |  | 1 | | ## RDW v2 | 17,735 | 3,010 | 0.170 | | | |
|  | | |  | 2 | | ## control v2 | 17,735 | 1,975 | 0.111 | | | |
|  | | | | | | | | | | | | |
|  | | |  |  | | |  | 95% CI | z | p |  |  |
|  | | |  | **Risk Difference** | | | 0.058 | (0.051, 0.066) | 15.812 | 0.000 |  |  |
|  | | |  | **Risk Ratio** | | | 1.524 | (1.446, 1.607) | N/A | N/A |  |  |
|  | | |  | **Odds Ratio** | | | 1.631 | (1.535, 1.734) | N/A | N/A |  |  |
|  | | | | | | | | | | | | |
|  | |  | | |  | | | | | | | |
|  | | **Kaplan - Meier survival analysis** | | | | | | | | | | |
|  | | |  | Cohort | | | Patients in cohort | Patients with outcome | Median survival (days) | Survival probability at end of time window | | |
|  | | |  | 1 | | ## RDW v2 | 17,735 | 3,010 | -- | 82.00% | | |
|  | | |  | 2 | | ## control v2 | 17,735 | 1,975 | -- | 88.09% | | |
|  | | | | | | | | | | | | |
|  | | |  |  | | | χ^2^ | df | p |  |  |  |
|  | | |  | **Log-Rank Test** | | | 247.151 | 1 | 0.000 |  |  |  |
|  | | | | | | | | | | | | |
|  | | |  |  | | | Hazard Ratio | 95% CI | χ^2^ | df | p | |
|  | | |  | **Hazard Ratio and Proportionality** | | | 1.570 | (1.484, 1.662) | 3.573 | 1 | 0.059 | |
|  | | | | | | | | | | | | |
|  | |  | | |  | | | | | | | |
| **8 urinary tract infection** | | | | | | | | | | | | |
|  | | **Risk analysis** | | | | | | | | | | |
|  |  | | | Cohort | | | Patients in cohort | Patients with outcome | Risk | | | |
|  | | |  | 1 | | ## RDW v2 | 17,735 | 769 | 0.043 | | | |
|  | | |  | 2 | | ## control v2 | 17,735 | 761 | 0.043 | | | |
|  | | | | | | | | | | | | |
|  | | |  |  | | |  | 95% CI | z | p |  |  |
|  | | |  | **Risk Difference** | | | 0.000 | (-0.004, 0.005) | 0.209 | 0.834 |  |  |
|  | | |  | **Risk Ratio** | | | 1.011 | (0.916, 1.115) | N/A | N/A |  |  |
|  | | |  | **Odds Ratio** | | | 1.011 | (0.913, 1.120) | N/A | N/A |  |  |
|  | | | | | | | | | | | | |
|  | |  | | |  | | | | | | | |
|  | | **Kaplan - Meier survival analysis** | | | | | | | | | | |
|  | | |  | Cohort | | | Patients in cohort | Patients with outcome | Median survival (days) | Survival probability at end of time window | | |
|  | | |  | 1 | | ## RDW v2 | 17,735 | 769 | -- | 95.45% | | |
|  | | |  | 2 | | ## control v2 | 17,735 | 761 | -- | 95.45% | | |
|  | | | | | | | | | | | | |
|  | | |  |  | | | χ^2^ | df | p |  |  |  |
|  | | |  | **Log-Rank Test** | | | 0.016 | 1 | 0.898 |  |  |  |
|  | | | | | | | | | | | | |
|  | | |  |  | | | Hazard Ratio | 95% CI | χ^2^ | df | p | |
|  | | |  | **Hazard Ratio and Proportionality** | | | 1.007 | (0.911, 1.113) | 5.314 | 1 | 0.021 | |
|  | | | | | | | | | | | | |
|  | |  | | |  | | | | | | | |
| **9 CRP** | | | | | | | | | | | | |
|  | | **Risk analysis** | | | | | | | | | | |
|  |  | | | Cohort | | | Patients in cohort | Patients with outcome | Risk | | | |
|  | | |  | 1 | | ## RDW v2 | 17,735 | 646 | 0.036 | | | |
|  | | |  | 2 | | ## control v2 | 17,735 | 495 | 0.028 | | | |
|  | | | | | | | | | | | | |
|  | | |  |  | | |  | 95% CI | z | p |  |  |
|  | | |  | **Risk Difference** | | | 0.009 | (0.005, 0.012) | 4.544 | 0.000 |  |  |
|  | | |  | **Risk Ratio** | | | 1.305 | (1.163, 1.464) | N/A | N/A |  |  |
|  | | |  | **Odds Ratio** | | | 1.317 | (1.169, 1.483) | N/A | N/A |  |  |
|  | | | | | | | | | | | | |
|  | |  | | |  | | | | | | | |
|  | | **Kaplan - Meier survival analysis** | | | | | | | | | | |
|  | | |  | Cohort | | | Patients in cohort | Patients with outcome | Median survival (days) | Survival probability at end of time window | | |
|  | | |  | 1 | | ## RDW v2 | 17,735 | 646 | -- | 96.21% | | |
|  | | |  | 2 | | ## control v2 | 17,735 | 495 | -- | 97.10% | | |
|  | | | | | | | | | | | | |
|  | | |  |  | | | χ^2^ | df | p |  |  |  |
|  | | |  | **Log-Rank Test** | | | 19.852 | 1 | 0.000 |  |  |  |
|  | | | | | | | | | | | | |
|  | | |  |  | | | Hazard Ratio | 95% CI | χ^2^ | df | p | |
|  | | |  | **Hazard Ratio and Proportionality** | | | 1.304 | (1.160, 1.466) | 4.802 | 1 | 0.028 | |
|  | | | | | | | | | | | | |
|  | |  | | |  | | | | | | | |

# Appendix A – Text Representation of the Cohorts Definition

This section lists all terms used in the definitions of the two cohorts.

### Query Criteria for Cohort 1 (query name: ## RDW v2)

Patients must have:
 Age (Age) (at least 18 years (most recent occurrence)).

All the following must be satisfied:

 Primary Sleeve (and) RYGB: The terms in this group occurred between Jan 1, 2010 and Dec 31, 2025
 Patients must have:
 any of the following:
 Laparoscopy, surgical, gastric restrictive procedure; with gastric bypass and Roux-en-Y gastroenterostomy (roux limb 150 cm or less) (UMLS:CPT:43644) (at least 18 years old at event); or
 Laparoscopy, surgical, gastric restrictive procedure; longitudinal gastrectomy (ie, sleeve gastrectomy) (UMLS:CPT:43775) (at least 18 years old at event).
 ZD: Any instance of ZD occurred within 3 months on or before any instance of Primary Sleeve (and) RYGB
 Patients must have:
 Erythrocyte distribution width [Ratio] (TNX:9008) (at least 14.60 %).
 Patients cannot have:
 Erythrocyte distribution width [Ratio] (TNX:9008) (between 11.50 and 14.50 %).


 Primary Sleeve (and) RYGB: The terms in this group occurred between Jan 1, 2010 and Dec 31, 2025
 Patients must have:
 any of the following:
 Laparoscopy, surgical, gastric restrictive procedure; with gastric bypass and Roux-en-Y gastroenterostomy (roux limb 150 cm or less) (UMLS:CPT:43644) (at least 18 years old at event); or
 Laparoscopy, surgical, gastric restrictive procedure; longitudinal gastrectomy (ie, sleeve gastrectomy) (UMLS:CPT:43775) (at least 18 years old at event).


 Primary Sleeve (and) RYGB: The terms in this group occurred between Jan 1, 2010 and Dec 31, 2025
 Patients must have:
 any of the following:
 Laparoscopy, surgical, gastric restrictive procedure; with gastric bypass and Roux-en-Y gastroenterostomy (roux limb 150 cm or less) (UMLS:CPT:43644) (at least 18 years old at event); or
 Laparoscopy, surgical, gastric restrictive procedure; longitudinal gastrectomy (ie, sleeve gastrectomy) (UMLS:CPT:43775) (at least 18 years old at event).
 Group 3B: Any instance of Group 3B occurred at least 1 day before any instance of Primary Sleeve (and) RYGB
 Patients cannot have:
 any of the following:
 End stage renal disease (UMLS:ICD10CM:N18.6); or
 Chronic kidney disease, stage 5 (UMLS:ICD10CM:N18.5); or
 Chronic kidney disease, stage 4 (severe) (UMLS:ICD10CM:N18.4); or
 Dependence on renal dialysis (UMLS:ICD10CM:Z99.2); or
 HIV Status (NAACCR:BIOM:3859); or
 Human immunodeficiency virus [HIV] disease (UMLS:ICD10CM:B20).


 Primary Sleeve (and) RYGB: The terms in this group occurred between Apr 1, 2010 and Dec 31, 2025
 Patients must have:
 any of the following:
 Laparoscopy, surgical, gastric restrictive procedure; with gastric bypass and Roux-en-Y gastroenterostomy (roux limb 150 cm or less) (UMLS:CPT:43644) (at least 18 years old at event); or
 Laparoscopy, surgical, gastric restrictive procedure; longitudinal gastrectomy (ie, sleeve gastrectomy) (UMLS:CPT:43775) (at least 18 years old at event).
 Group 4B: Any instance of Group 4B occurred within 1 month and 1 day before any instance of Primary Sleeve (and) RYGB
 Patients cannot have:
 any of the following:
 Severe sepsis (UMLS:ICD10CM:R65.2); or
 Other sepsis (UMLS:ICD10CM:A41); or
 Critical Care Services (UMLS:CPT:1013729); or
 COVID-19 (UMLS:ICD10CM:U07.1); or
 Acute kidney failure (UMLS:ICD10CM:N17); or
 Influenza and pneumonia (UMLS:ICD10CM:J09-J18).


 Primary Sleeve (and) RYGB: The terms in this group occurred between Jan 1, 2010 and Dec 31, 2025
 Patients must have:
 any of the following:
 Laparoscopy, surgical, gastric restrictive procedure; with gastric bypass and Roux-en-Y gastroenterostomy (roux limb 150 cm or less) (UMLS:CPT:43644) (at least 18 years old at event); or
 Laparoscopy, surgical, gastric restrictive procedure; longitudinal gastrectomy (ie, sleeve gastrectomy) (UMLS:CPT:43775) (at least 18 years old at event).


 Primary Sleeve (and) RYGB: The terms in this group occurred at any time
 Patients must have:
 any of the following:
 Laparoscopy, surgical, gastric restrictive procedure; with gastric bypass and Roux-en-Y gastroenterostomy (roux limb 150 cm or less) (UMLS:CPT:43644) (at least 18 years old at event); or
 Laparoscopy, surgical, gastric restrictive procedure; longitudinal gastrectomy (ie, sleeve gastrectomy) (UMLS:CPT:43775) (at least 18 years old at event).
 Group 6B: Any instance of Group 6B occurred on the same date as any instance of Primary Sleeve (and) RYGB
 Patients cannot have:
 Transfusion, blood or blood components (UMLS:CPT:36430).

### Query Criteria for Cohort 2 (query name: ## control v2)

Patients must have:
 Age (Age) (at least 18 years (most recent occurrence)).

All the following must be satisfied:

 Primary Sleeve (and) RYGB: The terms in this group occurred between Jan 1, 2010 and Dec 31, 2025
 Patients must have:
 any of the following:
 Laparoscopy, surgical, gastric restrictive procedure; with gastric bypass and Roux-en-Y gastroenterostomy (roux limb 150 cm or less) (UMLS:CPT:43644) (at least 18 years old at event); or
 Laparoscopy, surgical, gastric restrictive procedure; longitudinal gastrectomy (ie, sleeve gastrectomy) (UMLS:CPT:43775) (at least 18 years old at event).
 ZD: Any instance of ZD occurred within 3 months on or before any instance of Primary Sleeve (and) RYGB
 Patients must have:
 Erythrocyte distribution width [Ratio] (TNX:9008) (between 11.50 and 14.50 %).
 Patients cannot have:
 Erythrocyte distribution width [Ratio] (TNX:9008) (at least 14.60 %).


 Primary Sleeve (and) RYGB: The terms in this group occurred between Jan 1, 2010 and Dec 31, 2025
 Patients must have:
 any of the following:
 Laparoscopy, surgical, gastric restrictive procedure; with gastric bypass and Roux-en-Y gastroenterostomy (roux limb 150 cm or less) (UMLS:CPT:43644) (at least 18 years old at event); or
 Laparoscopy, surgical, gastric restrictive procedure; longitudinal gastrectomy (ie, sleeve gastrectomy) (UMLS:CPT:43775) (at least 18 years old at event).


 Primary Sleeve (and) RYGB: The terms in this group occurred between Jan 1, 2010 and Dec 31, 2025
 Patients must have:
 any of the following:
 Laparoscopy, surgical, gastric restrictive procedure; with gastric bypass and Roux-en-Y gastroenterostomy (roux limb 150 cm or less) (UMLS:CPT:43644) (at least 18 years old at event); or
 Laparoscopy, surgical, gastric restrictive procedure; longitudinal gastrectomy (ie, sleeve gastrectomy) (UMLS:CPT:43775) (at least 18 years old at event).
 Group 3B: Any instance of Group 3B occurred at least 1 day before any instance of Primary Sleeve (and) RYGB
 Patients cannot have:
 any of the following:
 End stage renal disease (UMLS:ICD10CM:N18.6); or
 Chronic kidney disease, stage 5 (UMLS:ICD10CM:N18.5); or
 Chronic kidney disease, stage 4 (severe) (UMLS:ICD10CM:N18.4); or
 Dependence on renal dialysis (UMLS:ICD10CM:Z99.2); or
 HIV Status (NAACCR:BIOM:3859); or
 Human immunodeficiency virus [HIV] disease (UMLS:ICD10CM:B20).


 Primary Sleeve (and) RYGB: The terms in this group occurred between Apr 1, 2010 and Dec 31, 2025
 Patients must have:
 any of the following:
 Laparoscopy, surgical, gastric restrictive procedure; with gastric bypass and Roux-en-Y gastroenterostomy (roux limb 150 cm or less) (UMLS:CPT:43644) (at least 18 years old at event); or
 Laparoscopy, surgical, gastric restrictive procedure; longitudinal gastrectomy (ie, sleeve gastrectomy) (UMLS:CPT:43775) (at least 18 years old at event).
 Group 4B: Any instance of Group 4B occurred within 1 month and 1 day before any instance of Primary Sleeve (and) RYGB
 Patients cannot have:
 any of the following:
 Severe sepsis (UMLS:ICD10CM:R65.2); or
 Other sepsis (UMLS:ICD10CM:A41); or
 Critical Care Services (UMLS:CPT:1013729); or
 COVID-19 (UMLS:ICD10CM:U07.1); or
 Acute kidney failure (UMLS:ICD10CM:N17); or
 Influenza and pneumonia (UMLS:ICD10CM:J09-J18).


 Primary Sleeve (and) RYGB: The terms in this group occurred between Jan 1, 2010 and Dec 31, 2025
 Patients must have:
 any of the following:
 Laparoscopy, surgical, gastric restrictive procedure; with gastric bypass and Roux-en-Y gastroenterostomy (roux limb 150 cm or less) (UMLS:CPT:43644) (at least 18 years old at event); or
 Laparoscopy, surgical, gastric restrictive procedure; longitudinal gastrectomy (ie, sleeve gastrectomy) (UMLS:CPT:43775) (at least 18 years old at event).


 Primary Sleeve (and) RYGB: The terms in this group occurred at any time
 Patients must have:
 any of the following:
 Laparoscopy, surgical, gastric restrictive procedure; with gastric bypass and Roux-en-Y gastroenterostomy (roux limb 150 cm or less) (UMLS:CPT:43644) (at least 18 years old at event); or
 Laparoscopy, surgical, gastric restrictive procedure; longitudinal gastrectomy (ie, sleeve gastrectomy) (UMLS:CPT:43775) (at least 18 years old at event).
 Group 6B: Any instance of Group 6B occurred on the same date as any instance of Primary Sleeve (and) RYGB
 Patients cannot have:
 Transfusion, blood or blood components (UMLS:CPT:36430).

# Appendix B – Text Representation of the Analysis Setup

This section contains the Index Event definition for each cohort.

The index event for Cohort 1 (query name: ## RDW v2) is defined as the following:

All the following must be satisfied:

 Primary Sleeve (and) RYGB: The terms in this group occurred between Jan 1, 2010 and Dec 31, 2025
 Patients must have:
 any of the following:
 Laparoscopy, surgical, gastric restrictive procedure; with gastric bypass and Roux-en-Y gastroenterostomy (roux limb 150 cm or less) (UMLS:CPT:43644) (at least 18 years old at event); or
 Laparoscopy, surgical, gastric restrictive procedure; longitudinal gastrectomy (ie, sleeve gastrectomy) (UMLS:CPT:43775) (at least 18 years old at event).
 ZD: Any instance of ZD occurred within 3 months on or before any instance of Primary Sleeve (and) RYGB
 Patients must have:
 Erythrocyte distribution width [Ratio] (TNX:9008) (at least 14.60 %).
 Patients cannot have:
 Erythrocyte distribution width [Ratio] (TNX:9008) (between 11.50 and 14.50 %).

The index event for Cohort 2 (query name: ## control v2) is defined as the following:

All the following must be satisfied:

 Primary Sleeve (and) RYGB: The terms in this group occurred between Jan 1, 2010 and Dec 31, 2025
 Patients must have:
 any of the following:
 Laparoscopy, surgical, gastric restrictive procedure; with gastric bypass and Roux-en-Y gastroenterostomy (roux limb 150 cm or less) (UMLS:CPT:43644) (at least 18 years old at event); or
 Laparoscopy, surgical, gastric restrictive procedure; longitudinal gastrectomy (ie, sleeve gastrectomy) (UMLS:CPT:43775) (at least 18 years old at event).
 ZD: Any instance of ZD occurred within 3 months on or before any instance of Primary Sleeve (and) RYGB
 Patients must have:
 Erythrocyte distribution width [Ratio] (TNX:9008) (between 11.50 and 14.50 %).
 Patients cannot have:
 Erythrocyte distribution width [Ratio] (TNX:9008) (at least 14.60 %).

# Appendix C – Text Representation of the Outcomes Definition

This analysis includes the following outcomes:

Hb<12
 Patients must have:
 Hemoglobin [Mass/volume] in Blood (TNX:9014) (at most 12.90 g/dL (most recent occurrence)).

ICU
 Patients must have:
 Critical Care Services (UMLS:CPT:1013729).

sepsis
 Patients must have:
 any of the following:
 Severe sepsis (UMLS:ICD10CM:R65.2); or
 Other sepsis (UMLS:ICD10CM:A41); or
 Streptococcal sepsis (UMLS:ICD10CM:A40).

RDW>14.5
 Patients must have:
 Erythrocyte distribution width [Ratio] (TNX:9008) (at least 14.60 % (most recent occurrence)).

pneumonia
 Patients must have:
 any of the following:
 Viral pneumonia, not elsewhere classified (UMLS:ICD10CM:J12); or
 Pneumonia due to Streptococcus pneumoniae (UMLS:ICD10CM:J13); or
 Pneumonia due to Hemophilus influenzae (UMLS:ICD10CM:J14); or
 Bacterial pneumonia, not elsewhere classified (UMLS:ICD10CM:J15); or
 Pneumonia due to other infectious organisms, not elsewhere classified (UMLS:ICD10CM:J16); or
 Pneumonia in diseases classified elsewhere (UMLS:ICD10CM:J17); or
 Pneumonia, unspecified organism (UMLS:ICD10CM:J18); or
 Pneumonia due to other specified organism (UMLS:ICD9CM:483).

SSI
 Patients must have:
 any of the following:
 Infection following a procedure, superficial incisional surgical site (UMLS:ICD10CM:T81.41); or
 Infection following a procedure, deep incisional surgical site (UMLS:ICD10CM:T81.42).

Ferritin<30
 Patients must have:
 Ferritin [Mass/volume] in Serum, Plasma or Blood (TNX:9042) (at most 29.90 ng/mL (most recent occurrence)).

urinary tract infection
 Patients must have:
 Urinary tract infection, site not specified (UMLS:ICD10CM:N39.0).

CRP
 Patients must have:
 C reactive protein [Mass/volume] in Serum, Plasma or Blood (TNX:9063) (at least 10.00 mg/L (most recent occurrence)).
